# Toward the future management of patients with CML and Ph + ALL: real-world safety insights from dasatinib pharmacovigilance

**DOI:** 10.3389/fmed.2025.1709089

**Published:** 2026-01-06

**Authors:** Zhen Lu, Guangbin Shang, Yingjian Zeng, Xiaonan Lu

**Affiliations:** 1 The Affiliated Hospital of Jiangxi University of Chinese Medicine, Nanchang, China; 2 Research Center for Differentiation and Development of Basic Theory of Traditional Chinese Medicine, Nanchang, China; 3 School of Chinese Medicine, Jiangxi University of Chinese Medicine, Nanchang, China

**Keywords:** adverse events, chronic myeloid leukemia, dasatinib, FAERS, pharmacovigilance, signal detection, tyrosine kinase inhibitor

## Abstract

**Background:**

Dasatinib, a second-generation BCR-ABL1 tyrosine kinase inhibitor, has transformed treatment for chronic myeloid leukemia and Philadelphia chromosome–positive acute lymphoblastic leukemia. However, its broad kinase inhibition leads to distinct adverse events (AEs), requiring systematic post-marketing surveillance.

**Objective:**

To evaluate dasatinib-associated AEs using the United States Food and Drug Administration Adverse Event Reporting System (FAERS) and identify potential safety signals.

**Methods:**

We extracted FAERS reports (Q1 2004–Q4 2024) listing dasatinib as the primary suspect drug, submitted by physicians or pharmacists. After data cleaning, AEs were coded using MedDRA terminology. Signal detection was performed with four disproportionality methods (ROR, PRR, BCPNN, MGPS). Time-to-onset was analyzed using Weibull models, with subgroup analyses by demographic and clinical characteristics.

**Results:**

Among 7,213 cases, respiratory disorders were the most prominent signal (ROR = 2.74). A total of 1,951 significant signals were identified, 113 confirmed by all algorithms. Strongest disproportionality signals included blast cell proliferation (ROR = 518.94), primary effusion lymphoma (ROR = 181.38), and allogeneic bone marrow transplantation therapy (ROR = 171.48). Common AEs were pleural effusion (*n* = 828, ROR = 35.87), hepatotoxicity (*n* = 262, ROR = 29.17), and fluid retention (*n* = 129, ROR = 14.49). Weibull analysis showed an early failure pattern (β = 0.64), with 25.6% of AEs within 30 days and 28.2% after 360 days. Subgroup analysis revealed pleural effusion across all demographics, while hematologic signals predominated in adults and elderly patients.

**Conclusion:**

This large FAERS analysis characterized known respiratory complications of dasatinib, particularly pleural effusion, and identified several rare but potentially relevant safety signals hepatotoxicity, cardiovascular events, and novel AEs such as alveolar proteinosis and cytomegalovirus-related complications. The early failure pattern underscores the need for intensive monitoring during initiation and continued vigilance during long-term therapy. These findings provide real-world, hypothesis-generating evidence to optimize safety management and inform future clinical investigations, and should be interpreted with caution in view of the inherent limitations of spontaneous reporting data.

## Introduction

Chronic myeloid leukemia (CML) and Philadelphia chromosome-positive acute lymphoblastic leukemia (Ph + ALL) are hematological malignancies driven by Breakpoint Cluster Region-Abelson murine leukemia viral oncogene homolog 1 (BCR-ABL1) fusion oncoprotein, which constitutively activates tyrosine kinase signaling and promotes malignant proliferation and survival (1, 2). CML is characterized by an initial chronic phase that, if untreated, progresses to an accelerated phase and a blast phase, both of which are associated with poor prognosis (3). Ph + ALL represents a subset of acute lymphoblastic leukemia with an adverse prognosis compared to BCR-ABL1-negative disease, due to its aggressive clinical course and high relapse rate despite intensive chemotherapy (4). The advent of molecular-targeted therapies has transformed the management of these disorders, improving remission rates and overall survival (5).

Dasatinib is a second-generation BCR-ABL1 tyrosine kinase inhibitor approved for treating chronic, accelerated, and blast-phase CML as well as Ph + ALL, particularly in patients who are resistant or intolerant to imatinib (6). In pivotal clinical trials, dasatinib achieved higher rates of complete cytogenetic response and major molecular response compared to imatinib in newly diagnosed chronic-phase CML, and it also induced durable remission in imatinib-resistant disease (7). However, the kinase inhibitory profile of dasatinib extends beyond BCR-ABL1 to include Src family kinases, c-KIT, platelet-derived growth factor receptor (PDGFR), and EphA2 (an ephrin-A family receptor), contributing to a distinct spectrum of AEs (8, 9). Common AEs observed in clinical studies include myelosuppression (neutropenia and thrombocytopenia), fluid retention (pleural effusion and peripheral edema), gastrointestinal disturbances, and dermatological reactions (10).

Less common, but serious toxicities include pulmonary arterial hypertension, cardiac dysfunction, and hemorrhagic events (the latter is linked, in part, to dasatinib-associated platelet dysfunction) (11, 12). These toxicities have prompted dose modifications, treatment interruptions, and, in rare cases, discontinuation, underscoring the need for systematic pharmacovigilance to characterize the real-world safety profiles (10).

The United States Food and Drug Administration Adverse Event Reporting System (FAERS) is a public database that collates spontaneous AE reports submitted by healthcare professionals, consumers, and manufacturers worldwide. FAERS supports post-marketing surveillance by enabling the detection of safety signals through disproportionality analysis and temporal trend assessment (13). Although FAERS is subject to underreporting, reporting biases, and lack of denominator data, it remains a cornerstone for hypothesis generation and signal detection, complementing clinical trial data and observational studies.

In this study, we used the FAERS database to systematically evaluate AEs reported for dasatinib, distinguishing reports submitted by internal medicine physicians from those submitted by pharmacists. We aimed to identify and characterize the spectrum of dasatinib-associated AEs in routine clinical practice, thereby informing risk-mitigation strategies and optimizing patient management.

## Materials and methods

### Data sources and processing

The quarterly FAERS data were obtained from the FDA website. Data cleaning and integration were conducted using R software (version 4.3.1). Reports were retained if the preferred term (PT) count was ≥3. Duplicate entries were removed; for reports with multiple AEs under the same CASE_ID, only the most recent FDA date (FDA_DT) and the higher primary_ID were retained. Records lacking essential information or detailed drug data were excluded. Reports without any coded adverse event term (exposure-only records) were also excluded before signal detection.

**FIGURE 1:**
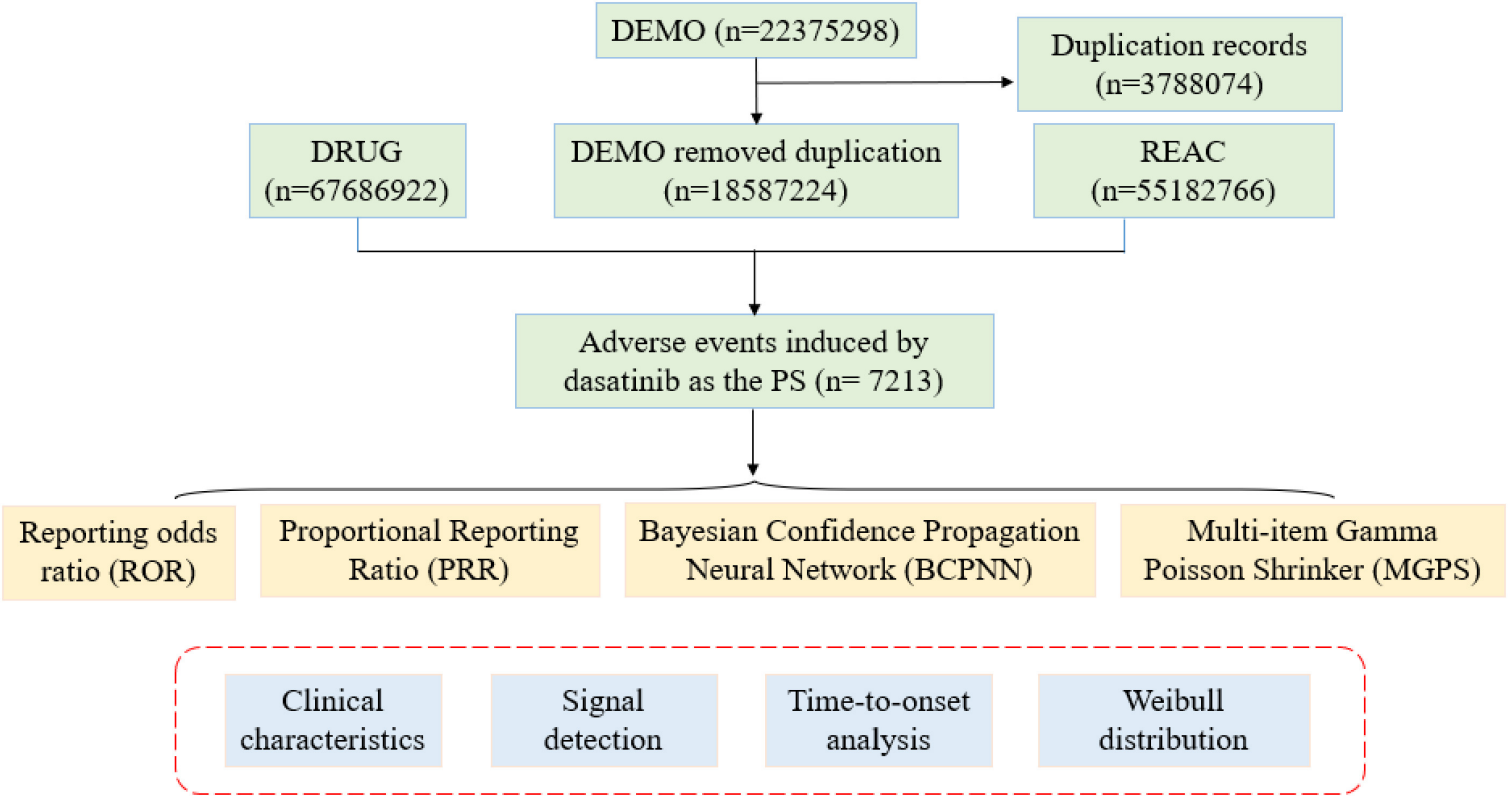
The flowchart of the entire study showing the selection of FAERS reports listing dasatinib as the primary suspect drug (Q1 2004–Q4 2024) and reported by physicians or pharmacists.

Adverse events were coded using the Medical Dictionary for Regulatory Activities to standardize the System Organ Class (SOC) and PT terminology. Reports between Q1 2004 and Q4 2024 were extracted and restricted to those listing dasatinib as a primary suspected drug. To improve reliability, only the cases submitted by physicians or pharmacists were included. The initial dataset contained 22,375,298 reports. After removing 3,788,074 duplicates, 7,213 unique cases were identified (Figure 1). Key demographic and clinical variables, including PRIMARYID, AGE, and GENDER scores, were extracted.

### Signal detection

A 2 × 2 contingency table was constructed to compare the observed frequencies of adverse events between the exposed and non-exposed groups (Supplementary Table 1). Disproportionality analyses were performed using four established methods: reporting odds ratio (ROR), proportional reporting ratio (PRR), Bayesian confidence propagation neural network (BCPNN), and multi-item gamma Poisson shrinker (MGPS). ROR is computationally simple and well suited to large-scale screening, PRR offers an intuitive assessment of disproportionality, BCPNN reduces false positives in small or rare-event datasets, and MGPS incorporates Bayesian shrinkage to address sparse data and multiple testing, making it particularly robust for long-term pharmacovigilance (14) (Supplementary Table 2). For primary signal detection, we predefined the following thresholds based on the 2 × 2 contingency table: for ROR and PRR, at least three reports (a ≥ 3) with the lower limit of the 95% confidence interval greater than 1; for BCPNN, a positive expectation of the information component [0 < E(IC)]; and for MGPS, a lower 95% confidence limit of the empirical Bayes geometric mean greater than 2 with at least one report (EBGM_05 > 2, a > 0) (Supplementary Table 2). Drug–event pairs that met the predefined thresholds of all four algorithms were included in the analysis. Time-to-onset (TTO) was calculated as TTO = AE onset date – dasatinib initiation date. Reports with erroneous entries (such as EVENT_DT earlier than START_DT) were excluded. Median values were used to summarize the timing of onset. A parametric Weibull model was fitted to the TTO distribution to characterize the overall time pattern of reporting; because FAERS does not provide consistent information on treatment line, cumulative exposure, or dose modifications, this analysis was intended to describe reporting dynamics rather than to define specific early- versus late-treatment phases. In addition, sensitivity analyses were performed by recalculating disproportionality metrics stratified by reporter role (physicians vs. pharmacists) and by the three main reporting countries (United States, Japan, and France).

## Results

### Baseline characteristics

**FIGURE 2:**
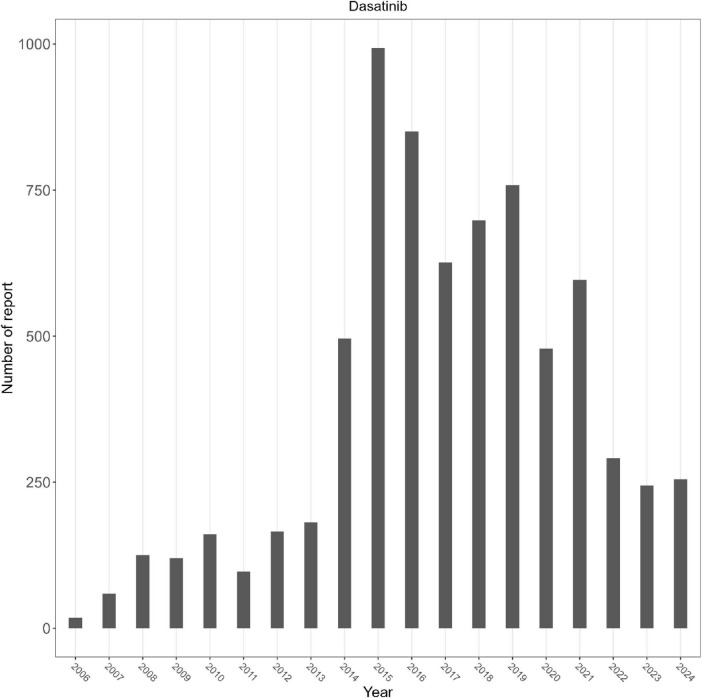
Number of dasatinib-associated AE reports per year.

Among the 7,213 included cases (Table 1), female patients accounted for 3,081 (42.7%) and male patients for 3,095 (42.9%). Weight data were largely missing (90.4%, *n* = 6,522). Among those with available information, 7.4% (*n* = 533) weighed 50–100 kg, 0.8% (*n* = 61) weighed < 50 kg, and 1.3% (*n* = 97) weighed > 100 kg. Patients aged 18–64.9 years represented the largest group (36.5%, *n* = 2,634), followed by those aged 65–85 years (19.9%, *n* = 1,436). Children (<18 years) accounted for 3.9% (*n* = 281), while patients > 85 years comprised 1.2% (*n* = 84). Serious cases accounted for 53.3% (*n* = 3,846), whereas non-serious cases comprised 46.7% (*n* = 3,367). Fatal outcomes were reported in 622 cases (8.6%), with 91.4% (*n* = 6,591) surviving. Most reports originated from the United States (71.1%, *n* = 5,126), followed by Japan (7.2%, *n* = 519) and France (3.1%, *n* = 226). A temporal trend showed peak reporting in 2015 (*n* = 993), with a subsequent decline in later years (Figure 2).

**TABLE 1:** Basic information on AE reports for dasatinib.

ID	Count	Percentage
Overall	7213	
**Sex**		
Female	3081	42.70%
Male	3095	42.90%
Missing	1037	14.40%
**Weight**		
<50 kg	61	0.80%
>100 kg	97	1.30%
50 ∼ 100 kg	533	7.40%
Missing	6522	90.40%
**Age**		
<18	281	3.90%
>85	84	1.20%
18 ∼ 64.9	2634	36.50%
65 ∼ 85	1436	19.90%
Missing	2778	38.50%
**Role**		
Pharmacist	3029	42.00%
Physician	4184	58.00%
**Serious cases**		
No	3367	46.70%
Yes	3846	53.30%
**Fatal cases**		
No	6591	91.40%
Yes	622	8.60%
**Report country (top 3)**		
The United States	5126	71.10%
Japan	519	7.20%
France	226	3.10%

### Signal detection findings

Using ROR, PRR, IC, and EBGM methods, dasatinib-related AEs were distributed across multiple SOCs. As shown in Table 2, the most frequently reported were general disorders and administration site conditions (*n* = 2,404; ROR 0.98; PRR 0.98; IC −0.03; EBGM 0.98), respiratory, thoracic and mediastinal disorders (*n* = 2,080; ROR 2.74; PRR 2.52; IC 1.33; EBGM 2.51), gastrointestinal disorders (*n* = 1,808; ROR 1.41; PRR 1.37; IC 0.45; EBGM 1.37) and nervous system disorders (*n* = 999; ROR 0.74; PRR 0.76; IC −0.40; EBGM 0.76). Respiratory system signals (ROR = 2.74) were the most prominent, warranting clinical attention. Although general disorders accounted for the highest number of reports, their disproportionality indices were not significant, suggesting non-specific associations rather than drug-specific risks.

**TABLE 2:** Disproportionality analysis of dasatinib-related AEs by SOC.

SOC name	Case number	ROR (95% CI)	PRR (XX)	EBGM (EBGM05)	IC (IC025)
General disorders and administration site conditions	2404	0.98 (0.94–1.02)	0.98 (0.87)	0.98 (0.94)	−0.03 (−0.09)
Respiratory, thoracic and mediastinal disorders	2080	2.74 (2.62–2.87)	2.52 (1996.21)	2.51 (2.4)	1.33 (1.26)
Gastrointestinal disorders	1808	1.41 (1.35–1.48)	1.37 (194.25)	1.37 (1.3)	0.45 (0.38)
Nervous system disorders	999	0.74 (0.7–0.79)	0.76 (83.99)	0.76 (0.71)	−0.4 (−0.49)
Injury, poisoning and procedural complications	987	0.63 (0.59–0.68)	0.66 (196.94)	0.66 (0.61)	−0.61 (−0.7)
Investigations	985	0.87 (0.82–0.93)	0.88 (16.64)	0.88 (0.83)	−0.18 (−0.28)
Skin and subcutaneous tissue disorders	886	1.04 (0.97–1.11)	1.03 (1.03)	1.03 (0.97)	0.05 (−0.05)
Infections and infestations	765	0.8 (0.75–0.87)	0.81 (34.59)	0.81 (0.76)	−0.3 (−0.4)
Blood and lymphatic system disorders	693	1.53 (1.42–1.65)	1.51 (121.82)	1.51 (1.4)	0.59 (0.48)
Cardiac disorders	654	1.14 (1.06–1.24)	1.14 (11.48)	1.14 (1.05)	0.19 (0.07)
Neoplasms benign, malignant and unspecified (incl cysts and polyps)	629	1.19 (1.1–1.29)	1.18 (18.33)	1.18 (1.09)	0.24 (0.12)
Musculoskeletal and connective tissue disorders	613	0.8 (0.74–0.87)	0.81 (29.67)	0.81 (0.74)	−0.31 (−0.43)
Surgical and medical procedures	476	2.52 (2.3–2.76)	2.47 (420.77)	2.47 (2.25)	1.3 (1.16)
Metabolism and nutrition disorders	406	0.97 (0.88–1.08)	0.98 (0.26)	0.98 (0.88)	−0.04 (−0.18)
Hepatobiliary disorders	379	1.65 (1.49–1.83)	1.64 (95.63)	1.64 (1.48)	0.71 (0.56)
Vascular disorders	260	0.67 (0.6–0.76)	0.68 (40.09)	0.68 (0.6)	−0.56 (−0.73)
Eye disorders	246	0.8 (0.7–0.9)	0.8 (12.76)	0.8 (0.7)	−0.32 (−0.51)
Psychiatric disorders	236	0.3 (0.27–0.34)	0.31 (373.56)	0.31 (0.28)	−1.68 (−1.86)
Renal and urinary disorders	224	0.65 (0.57–0.74)	0.66 (41.24)	0.66 (0.58)	−0.61 (−0.8)
Immune system disorders	96	0.45 (0.37–0.55)	0.45 (65.23)	0.45 (0.37)	−1.15 (−1.44)
Reproductive system and breast disorders	83	0.82 (0.66–1.02)	0.83 (3.09)	0.83 (0.67)	−0.28 (−0.59)
Ear and labyrinth disorders	52	0.89 (0.68–1.17)	0.89 (0.66)	0.89 (0.68)	−0.16 (−0.56)
Pregnancy, puerperium and perinatal conditions	46	0.58 (0.43–0.77)	0.58 (14.28)	0.58 (0.43)	−0.79 (−1.2)
Congenital, familial and genetic disorders	40	0.73 (0.54–1)	0.73 (3.95)	0.73 (0.54)	−0.45 (−0.89)
Social circumstances	23	0.46 (0.3–0.69)	0.46 (14.93)	0.46 (0.3)	−1.13 (−1.69)
Product issues	22	0.1 (0.07–0.15)	0.1 (173.67)	0.1 (0.07)	−3.27 (−3.82)
Endocrine disorders	18	0.32 (0.2–0.51)	0.32 (25.46)	0.32 (0.2)	−1.62 (−2.24)

SOC, System Organ Class; ROR, reporting odds ratio; PRR, proportional reporting ratio; EBGM, empirical Bayes geometric mean; EBGM05, lower 5% one-sided confidence limit of EBGM; IC, information component; IC025, lower end of the 95% credibility interval of IC.

At the PT level, 1,951 dasatinib-related AE signals were identified, of which 113 signals were validated by all four algorithms. The detected signals encompassed a broad range of clinical manifestations, predominantly involving hematologic malignancy progression, pleural/pulmonary complications, transplantation outcomes, infectious events, and cardiovascular/metabolic effects. The three strongest signals by ROR ranking were: blast cell proliferation (*n* = 11; ROR 518.94; PRR 518.59; IC 8.39; EBGM 334.93), primary effusion lymphoma (*n* = 5; ROR 181.38; PRR 181.33; IC 7.25; EBGM 152.24) and allogeneic bone marrow transplantation therapy (*n* = 4; ROR 171.48; PRR 171.43; IC 7.18; EBGM 145.21). By absolute case counts, the most frequently reported PTs were: pleural effusion (*n* = 828; ROR 35.87; PRR 34.08; IC 5.04; EBGM 32.92), hepatotoxicity (*n* = 262; ROR 29.17; PRR 28.72; IC 4.8; EBGM 27.9) and fluid retention (*n* = 129; ROR 14.49; PRR 14.38; IC 3.83; EBGM 14.18).

In addition to these core adverse events, the analysis also identified several signals related to serosal effusions and respiratory system disorders, such as polyserositis (ROR = 21.34), chylothorax (ROR = 169.55), hydrothorax (ROR = 11.68), and alveolar proteinosis (ROR = 13.22), indicating the broad impact of dasatinib on the pleural–pulmonary environment. Furthermore, AEs associated with infections and immune function were observed, including cytomegalovirus enterocolitis (ROR = 16.07), cytomegalovirus colitis (ROR = 10.98), and neutrophilic dermatosis (23.88), some of which showed high signal strength and warrant clinical attention. Notably, this study not only confirmed well-documented AEs already listed on the dasatinib product label–such as pleural effusion (ROR = 35.87), fluid retention (ROR = 14.49), and hepatotoxicity (ROR = 29.17)–but also revealed more specific or uncommon manifestations, including skin hypopigmentation (ROR = 12.70) and skin depigmentation (ROR = 11.03). Beyond the known risks of myelosuppression and transplantation-related complications, the analysis also identified emerging clinically relevant events, such as hydrops foetalis (ROR = 14.43), peripheral artery stenosis (ROR = 16.61), and ejection fraction abnormal (ROR = 10.28). After excluding PTs attributable to underlying disease progression or treatment-related procedures, several rare but potentially important signals (e.g., alveolar proteinosis, drug tolerance decreased) were identified (Table 3). These findings deserve further validation through clinical and mechanistic research.

**TABLE 3:** Disproportionality analysis of dasatinib-related AEs by PT.

PT	Case number	ROR (95% CI)	PRR (XX)	EBGM (EBGM05)	IC (IC025)
Blast cell proliferation	11	518.94 (248.61–1083.23)	518.59 (3666.15)	334.93 (160.46)	8.39 (2.56)
Primary effusion lymphoma	5	181.38 (69.64–472.41)	181.33 (752.03)	152.24 (58.45)	7.25 (1.25)
Allogenic bone marrow transplantation therapy	4	171.48 (59.08–497.67)	171.43 (573.49)	145.21 (50.04)	7.18 (0.88)
Chylothorax	40	169.55 (121.05–237.47)	169.13 (5668.69)	143.56 (102.5)	7.17 (4.52)
Philadelphia chromosome positive	31	134.96 (92.6–196.68)	134.7 (3599.71)	117.99 (80.96)	6.88 (4.12)
Trisomy 8	3	108.82 (32.93–359.55)	108.8 (287.27)	97.64 (29.55)	6.61 (0.42)
Aspiration pleural cavity	19	92.45 (57.7–148.13)	92.35 (1563.64)	84.2 (52.55)	6.4 (3.35)
Lymphoid tissue hyperplasia	13	86.39 (48.95–152.48)	86.32 (1004.38)	79.17 (44.85)	6.31 (2.78)
Chronic myeloid leukemia transformation	9	57.76 (29.46–113.24)	57.73 (472.77)	54.46 (27.78)	5.77 (2.16)
Chronic myeloid leukemia recurrent	8	56.32 (27.59–114.96)	56.29 (410)	53.18 (26.05)	5.73 (1.97)
Cytogenetic analysis abnormal	22	53.12 (34.57–81.64)	53.05 (1063.75)	50.28 (32.72)	5.65 (3.38)
Exposure via body fluid	20	43.5 (27.78–68.13)	43.45 (792.97)	41.58 (26.55)	5.38 (3.18)
Pleural effusion	828	35.87 (33.41–38.51)	34.08 (25696.1)	32.92 (30.66)	5.04 (4.88)
Blast crisis in myelogenous leukemia	12	29.88 (16.81–53.09)	29.85 (324.39)	28.97 (16.3)	4.86 (2.39)
Hepatotoxicity	262	29.17 (25.77–33.02)	28.72 (6805.36)	27.9 (24.65)	4.8 (4.48)
Bone marrow transplant	13	29.14 (16.78–50.61)	29.12 (342.38)	28.27 (16.28)	4.82 (2.48)
Acquired gene mutation	6	27.21 (12.08–61.27)	27.2 (147.17)	26.46 (11.75)	4.73 (1.4)
Enterocolitis haemorrhagic	21	25.98 (16.84–40.1)	25.95 (490.31)	25.28 (16.38)	4.66 (2.96)
Prescribed underdose	119	25.93 (21.6–31.13)	25.75 (2756.05)	25.09 (20.9)	4.65 (4.12)
Neutrophilic dermatosis	4	23.88 (8.85–64.41)	23.87 (85.49)	23.31 (8.64)	4.54 (0.78)
Gene mutation identification test positive	4	22.32 (8.28–60.17)	22.32 (79.56)	21.82 (8.1)	4.45 (0.77)
Blast cell crisis	3	22.28 (7.09–70)	22.27 (59.55)	21.78 (6.93)	4.45 (0.35)
Polyserositis	5	21.34 (8.79–51.78)	21.33 (94.75)	20.88 (8.61)	4.38 (1.08)
Minimal residual disease	4	19.96 (7.41–53.74)	19.96 (70.53)	19.56 (7.27)	4.29 (0.75)
Gene mutation	10	19.25 (10.29–36.02)	19.24 (169.49)	18.88 (10.09)	4.24 (1.97)
Pleurisy	42	18.24 (13.44–24.77)	18.2 (669.84)	17.87 (13.17)	4.16 (3.24)
Hyperplasia	6	17.15 (7.65–38.46)	17.14 (89.58)	16.86 (7.52)	4.08 (1.26)
Peripheral artery stenosis	5	16.61 (6.86–40.21)	16.6 (72.04)	16.33 (6.74)	4.03 (1.01)
Cytomegalovirus enterocolitis	7	16.07 (7.61–33.92)	16.06 (97.2)	15.81 (7.49)	3.98 (1.44)
Lymphocytosis	17	15.68 (9.71–25.34)	15.67 (229.65)	15.43 (9.55)	3.95 (2.41)
Transplant	9	15.61 (8.08–30.16)	15.6 (120.97)	15.36 (7.95)	3.94 (1.73)
Acute lymphocytic leukemia recurrent	17	15.35 (9.51–24.8)	15.34 (224.24)	15.11 (9.35)	3.92 (2.4)
Fluid retention	129	14.49 (12.17–17.25)	14.38 (1582.44)	14.18 (11.91)	3.83 (3.43)
Hydrops foetalis	3	14.43 (4.61–45.15)	14.43 (36.94)	14.23 (4.55)	3.83 (0.27)
Chronic myeloid leukemia	24	13.73 (9.17–20.54)	13.71 (278.69)	13.52 (9.04)	3.76 (2.59)
Pericardial effusion	115	13.49 (11.22–16.23)	13.4 (1301.97)	13.23 (11)	3.73 (3.31)
Red blood cell abnormality	3	13.35 (4.27–41.71)	13.34 (33.78)	13.17 (4.21)	3.72 (0.25)
Alveolar proteinosis	3	13.22 (4.23–41.32)	13.22 (33.41)	13.05 (4.17)	3.71 (0.24)
Skin hypopigmentation	4	12.7 (4.74–34.07)	12.7 (42.54)	12.54 (4.68)	3.65 (0.62)
Pulmonary edema	155	11.81 (10.07–13.85)	11.71 (1500.72)	11.58 (9.87)	3.53 (3.2)
Hydrothorax	4	11.68 (4.36–31.31)	11.68 (38.57)	11.55 (4.31)	3.53 (0.59)
Therapy change	30	11.39 (7.94–16.33)	11.37 (280.38)	11.25 (7.84)	3.49 (2.56)
Pulmonary hypertension	84	11.08 (8.93–13.75)	11.03 (757.6)	10.91 (8.8)	3.45 (2.97)
Skin depigmentation	4	11.03 (4.12–29.56)	11.03 (36.05)	10.91 (4.07)	3.45 (0.57)
Cytomegalovirus colitis	9	10.98 (5.69–21.19)	10.98 (80.69)	10.86 (5.63)	3.44 (1.53)
Platelet transfusion	4	10.9 (4.07–29.22)	10.9 (35.56)	10.79 (4.03)	3.43 (0.57)
Gastrostomy	8	10.39 (5.18–20.87)	10.39 (67.15)	10.29 (5.12)	3.36 (1.37)
Ejection fraction abnormal	4	10.28 (3.84–27.54)	10.28 (33.14)	10.18 (3.8)	3.35 (0.54)
Stem cell transplant	8	10.17 (5.07–20.42)	10.17 (65.41)	10.07 (5.02)	3.33 (1.36)
Drug tolerance decreased	6	9.54 (4.27–21.33)	9.54 (45.41)	9.45 (4.23)	3.24 (1)

PT, preferred term; ROR, reporting odds ratio; PRR, proportional reporting ratio; EBGM, empirical Bayes geometric mean; EBGM05, lower 5% one-sided confidence limit of EBGM; IC, information component; IC025, lower end of the 95% credibility interval of IC. PTs clearly reflecting disease progression or treatment procedures (e.g., blast cell proliferation, CML transformation, bone marrow/stem cell transplantation) are listed for completeness but are not interpreted as direct dasatinib-induced AEs.

### Subgroup analyses

In subgroup analyses stratified by gender, age, and body weight, pleural effusion consistently emerged as the most prominent signal across all categories. Hematologic and cytogenetic events such as blast cell proliferation and Philadelphia chromosome positive were predominantly enriched among adults, elderly patients, and male populations. In contrast, children and low-weight patients exhibited some distinctive signals, including hemorrhagic enterocolitis, prescribed underdose, and gastrointestinal hemorrhage. Patients in the medium- and high-weight groups more frequently reported cardiopulmonary events, such as pericardial effusion, pulmonary hypertension, and pulmonary edema. Overall, these findings suggest that while fluid retention events (e.g., pleural effusion) are common across all populations, the spectrum of dasatinib-related adverse events varies according to demographic and clinical characteristics; however, these apparent differences may also reflect variation in prescribing patterns and reporting behavior between subgroups (Supplementary Tables 3–11).

### TTO analysis

**FIGURE 3:**
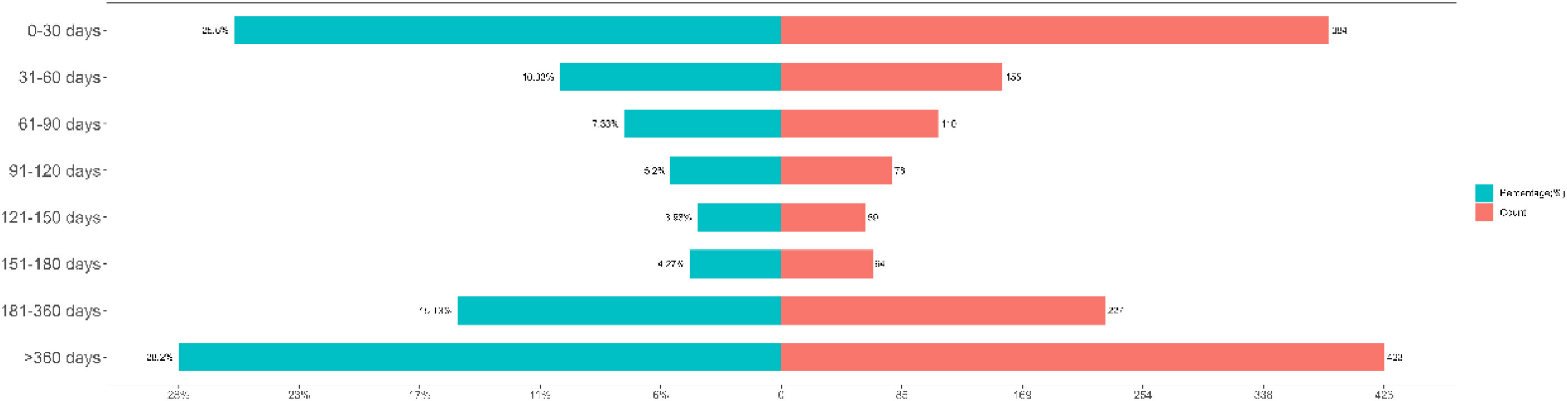
Distribution of the time to onset (TTO) of dasatinib-associated AEs.

A total of 1,500 reports documented the onset time of AEs and were included in the analysis. The results showed that 28.2% (*n* = 423) of AEs occurred after 360 days of treatment initiation. The distribution across other time intervals was as follows 0–30 days (25.6%, *n* = 384), 31–60 days (10.3%, *n* = 155), 61–90 days (7.3%, *n* = 110), 91–120 days (5.2%, *n* = 78), 121–150 days (3.9%, *n* = 59), 151–180 days (4.3%, *n* = 64), 181–360 days (15.1%, *n* = 227) (Figure 3). The mean time to onset for dasatinib-related AEs was 346.1 days, with a median onset time of 131.5 days and an interquartile range (IQR) of 29–413 days (Table 4).

**TABLE 4:** Time to onset analysis (days).

Mean	Median	Interquartile range
346.1	131.5	29–413

A Weibull distribution model was fitted to characterize the time-to-onset pattern. The estimated scale parameter (α) was 244.65 (95% CI: 224.16–265.15), and the shape parameter (β) was 0.64 (95% CI: 0.61–0.66). Since the 95% CI of β was entirely below 1, the time-to-onset of dasatinib-related AEs was classified as an early failure type, indicating that these events were more likely to occur during the early phase of treatment (Table 5). Because information on treatment line, cumulative exposure and dose modification is not captured in FAERS, this Weibull model should be regarded as a descriptive summary of reporting patterns rather than a formal survival analysis.

**TABLE 5:** Weibull parameters for AEs onset.

Scale parameter: α (95% CI)	Shape parameter: β (95% CI)	Type
244.65 (224.16–265.15)	0.64 (0.61–0.66)	Early failure

CI, confidence interval.

### Sensitivity analyses

To explore potential reporting bias, we performed sensitivity analyses stratified by reporter type (physicians vs. pharmacists) and by the three main reporting countries (the United States, Japan and France). Across all strata, pleural effusion remained the most prominent dasatinib-associated event, together with persistent signals for pulmonary edema, fluid retention, pericardial effusion and pulmonary hypertension. Hepatotoxicity showed a particularly strong signal in physician-reported and U.S. cases, whereas Japanese and French reports highlighted several rare but high-magnitude serosal and respiratory events, such as chylothorax and pulmonary arterial hypertension, albeit based on few cases. Overall, these findings indicate that the core pulmonary and hepatobiliary safety profile of dasatinib is robust to differences in reporter type and geographic origin (Supplementary Tables 12–16).

## Discussion

### Major findings and clinical implications

This comprehensive pharmacovigilance study of 7,213 dasatinib-related AEs from the FAERS database identified several critical safety signals that warrant clinical attention and mechanistic exploration. Our analysis identified 113 statistically significant PTs using rigorous four-algorithm screening, with respiratory system disorders emerging as the most prominent safety concern (ROR = 2.74). These findings not only confirm well-established adverse effects, but also highlight previously underreported safety signals that may have significant clinical implications for optimizing dasatinib therapy.

Pleural effusion was the most frequently reported AE (*n* = 828, ROR = 35.87), which aligns with established clinical knowledge and reinforces the critical importance of pulmonary monitoring during dasatinib therapy (14–16), with clinicians keeping a low threshold for evaluating new or worsening dyspnea, cough, or unexplained weight gain. However, the identification of novel signals, such as blast cell proliferation (ROR = 518.94), primary effusion lymphoma (ROR = 181.38), and allogeneic bone marrow transplantation therapy (ROR = 171.43), should be interpreted with caution. These findings require careful clinical interpretation because they may represent either true drug-related toxicities or confounding by indication, given the underlying malignant conditions being treated (17, 18).

Overall, these patterns support routine baseline and periodic assessment of respiratory symptoms, liver function, and cardiovascular status, especially in patients with pre-existing cardiopulmonary or hepatic comorbidities.

Temporal analysis, revealing an early failure pattern (Weibull β = 0.64, 95% CI: 0.61–0.66), provides crucial insights into clinical monitoring strategies. With 25.6% of AEs occurring within the first 30 days and a significant proportion (28.2%) occurring after more than 360 days, a bimodal surveillance approach is necessary. Early onset events likely reflect acute toxicities related to initial drug exposure, whereas late-onset events may indicate cumulative, dose-dependent effects or long-term off-target consequences (19–21).

To facilitate clinical interpretation, key safety signals and their main monitoring focus are briefly summarized in Table 6; these points are intended as pragmatic reminders rather than formal management recommendations.

**TABLE 6:** Key dasatinib-related safety signals and main monitoring focus.

Signal	Key concern	Monitoring focus
Pleural and other serosal effusions	Dyspnea, cough, chest discomfort, fluid overload	Baseline cardiopulmonary history; assess symptoms, weight and peripheral edema at routine visits
Pulmonary hypertension/ pulmonary edema	Progressive exertional dyspnea, right heart strain	Assess cardiovascular risk; symptom-triggered echocardiography where available
Hepatotoxicity	Asymptomatic liver enzyme elevation to clinically significant liver injury	Baseline liver function tests; periodic monitoring in the first months and in patients with pre-existing liver disease
Cardiovascular events	Heart failure, reduced ejection fraction, arterial events	Baseline cardiovascular risk assessment; monitor for chest pain, dyspnea and decreased exercise tolerance
Infections including CMV-related events	Opportunistic infections in immunocompromised/ post-transplant patients	Vigilance for fever and gastrointestinal symptoms; early diagnostic work-up in high-risk patients
Hematologic progression/disease-related events	Disease progression or treatment failure rather than pure drug toxicity	Regular hematologic and molecular response assessment according to CML/Ph + ALL guidelines

### Mechanistic insights into dasatinib-related AEs

#### Respiratory and pleural complications

The predominance of respiratory system disorders in our analysis reflects the complex interaction between dasatinib and pulmonary physiology. Pleural effusion, the most commonly reported AE, results from the inhibition of PDGFR and Src family kinases, leading to increased vascular permeability and lymphatic drainage abnormalities (22, 23). The mechanism involves disruption of endothelial barrier function through interference with PDGFR-mediated signaling pathways, which are crucial for maintaining vascular integrity. Additionally, the off-target effects of dasatinib on lymphatic drainage may contribute to fluid accumulation in serous cavities (24, 25).

Identifying chylothorax, hydrothorax, and polyserositis signals suggests a broader serosal involvement beyond simple pleural effusion. Despite being rare, chylothorax represents a more severe manifestation involving lymphatic system disruption, potentially through the effects of dasatinib on lymphatic endothelial cells or thoracic duct integrity (11). The mechanisms underlying these complications remain incompletely understood but may involve alterations in lymphangiogenesis or lymphatic vessel permeability via Src kinase pathway modulation (26).

Alveolar proteinosis, another notable respiratory signal in our analysis, suggests a potential interference with alveolar macrophage function or surfactant metabolism. This effect could result from the impact of dasatinib on macrophage polarization and clearance functions, given the influence of the drug on immune cell signaling pathways (27, 28). The clinical significance of this finding requires further investigation, as alveolar proteinosis can lead to progressive respiratory failure if left unrecognized.

#### Hepatotoxicity mechanisms

Hepatotoxicity emerged as the second most frequent AE by absolute count (*n* = 262, ROR = 29.17), reflecting multiple potential mechanisms of liver injury. Dasatinib-induced hepatotoxicity appears to involve disruption of bile acid homeostasis through inhibition of the bile salt export pump (BSEP), leading to intracellular bile acid accumulation and hepatocyte toxicity (29, 30). This mechanism is supported by studies demonstrating competitive inhibition of BSEP by dasatinib, with further elevation of serum biomarkers for hepatotoxicity in 25%–50% of patients (31).

Moreover, dasatinib may induce oxidative stress and mitochondrial dysfunction, thereby triggering autophagy as a protective mechanism. Research has demonstrated that dasatinib activates p38 signaling pathways, which modulate oxidative stress-related liver injury and autophagy (32, 33). The activation of autophagy appears to serve as a cellular defense mechanism, as autophagy inhibition can exacerbate dasatinib-induced liver failure (34). Furthermore, dasatinib treatment has been associated with increased cytochrome P450 7A1 (CYP7A1) expression, leading to enhanced bile acid synthesis and potential hepatotoxic accumulation (35).

The involvement of immune-mediated mechanisms in dasatinib hepatotoxicity has been suggested by studies demonstrating significant lymphocyte infiltration in liver tissue, including CD3+, CD4+, CD8+, and CD20+ cells (36). This inflammatory response may contribute to liver injury through cytokine-mediated pathways and represents a potential therapeutic target.

#### Hematologic and oncologic signals

Identifying blast cell proliferation as the strongest signal (ROR = 518.94) requires careful interpretation within the context of the therapeutic indication of dasatinib for hematologic malignancies. This signal may represent disease progression, treatment resistance, or potentially paradoxical effects of dasatinib on blast cell populations. In Philadelphia chromosome–positive leukemia, dasatinib functions primarily through BCR-ABL kinase inhibition; however, its broad kinase inhibitory profile may have unintended consequences on normal hematopoietic cell populations (37, 38).

Primary effusion lymphoma, another highly significant sign, is a rare but serious potential complication. This finding suggests possible interaction between dasatinib and viral-associated lymphoproliferative disorders, particularly given the immunomodulatory effects of dasatinib. This mechanism may involve alterations in immune surveillance or direct effects on lymphocyte populations (39).

The indication for allogeneic bone marrow transplantation therapy likely reflects the clinical context in which dasatinib is used because many patients with advanced hematologic malignancies may require transplantation. However, it also raises questions about the potential interactions between dasatinib and transplant-related complications, including graft-versus-host disease and transplant-related toxicities (40).

#### Cardiovascular and vascular effects

Our analysis revealed several cardiovascular signals, including peripheral artery stenosis and ejection fraction abnormalities, consistent with emerging concerns regarding tyrosine kinase inhibitor-associated vascular toxicities. Unlike nilotinib and ponatinib, which are more strongly associated with arterial occlusive events, dasatinib appears to have a relatively lower incidence of peripheral arterial disease (7, 41). However, identifying these signals suggests that cardiovascular monitoring remains important, particularly in patients with pre-existing cardiovascular risk factors.

The mechanism of dasatinib-induced cardiovascular effects may involve the off-target inhibition of kinases, which are important for endothelial function and vascular homeostasis. Src family kinases play crucial roles in endothelial cell signaling and vascular integrity, and their inhibition by dasatinib may contribute to endothelial dysfunction (42). Besides, the effects of dasatinib on nitric oxide signaling and inflammatory pathways may contribute to vascular complications (43).

#### Dermatologic and hypopigmentation effects

Identifying skin hypopigmentation and depigmentation signals provides insight into the effects of dasatinib on melanocyte function and pigment production. This effect is likely due to the inhibition of c-Kit signaling by the drug, which is essential for melanocyte survival and melanin synthesis (44). The mechanism involves the disruption of stem cell factor/c-Kit signaling pathways that regulate melanocyte development, migration, and pigment production.

Despite being generally cosmetic, these dermatological effects can be distressing to patients and may have broader effects on stem cell populations. The reversibility of these effects upon drug discontinuation suggests that the underlying melanocyte population remains intact with functional inhibition, rather than cellular destruction (45).

#### Novel and unexpected signals

The several unexpected signals identified in our analysis warrant further investigation. Despite being rare, hydrops fetalis raises significant concerns regarding dasatinib use during pregnancy and suggests potential teratogenic effects. The mechanism may involve interference with fetal cardiovascular development or fluid balance regulation, although the rarity of this event limits the current mechanistic understanding (46).

A decrease in drug tolerance is an interesting pharmacodynamic signal that may reflect the development of cellular resistance mechanisms or pharmacokinetic alterations that affect drug absorption or metabolism. This could involve the upregulation of efflux pumps, metabolic enzyme induction, or cellular adaptation mechanisms that reduce drug sensitivity over time (47).

Identifying cytomegalovirus-related complications (CMV enterocolitis and CMV colitis) suggests that the immunosuppressive effects of dasatinib may predispose patients to opportunistic infections. This effect is likely due to the impact of the drug on T cell function and immune surveillance, potentially through Src family kinase inhibition in the immune cells (48).

### Subgroup analysis and population-specific considerations

Subgroup analyses revealed important demographic and clinical variations in the adverse event profiles. The consistent appearance of pleural effusion across all demographic strata confirms that it is a universal concern that requires vigilant monitoring, regardless of patient characteristics. However, the enrichment of hematological signals (blast cell proliferation, Philadelphia chromosome-positive) in adult, elderly, and male patients suggests potential age- and sex-related differences in drug sensitivity or disease progression patterns.

The distinctive signals observed in pediatric and low-weight patients, including hemorrhagic enterocolitis and gastrointestinal hemorrhage, highlight the need for age-specific monitoring. These differences may reflect developmental variations in drug metabolism, organ sensitivity, or the underlying disease characteristics. The tendency of the medium- and high-weight groups to report more cardiopulmonary events suggests potential dose–weight relationships that may require therapeutic drug monitoring or dose optimization strategies.

### Temporal patterns and clinical monitoring implications

Weibull distribution analysis revealed an early failure pattern (β = 0.64), which may help refine clinical monitoring protocols. The high proportion of early onset events (25.6% within 30 days) supports intensive monitoring during treatment initiation, whereas the significant late-onset component (28.2% after 360 days) indicates the need for sustained vigilance throughout therapy.

This bimodal pattern suggests different underlying mechanisms of early and late AEs. Early events likely represent acute toxicities related to initial drug exposure and rapid pharmacological effects, although contributions from pre-existing comorbidities, more advanced or resistant disease, and baseline organ dysfunction cannot be excluded. Late events may reflect cumulative toxicities, organ dysfunction, or the development of secondary complications. A median time to onset of 131.5 days provides a useful benchmark for clinical monitoring, suggesting that most events occur within the first 4–5 months of therapy, but these time windows should be interpreted as hypothesis-generating given the limited treatment-history data in FAERS.

### Comparison with existing clinical practice

Our findings are largely consistent with established clinical knowledge regarding dasatinib safety profiles, while providing measures of disproportionality (signal strength) and novel signal identification. The prominence of pleural effusion aligns with clinical trial data demonstrating incidence rates of 10%–35% in patients treated with dasatinib (49). From a class perspective, pleural and other serosal effusions are more characteristic of dasatinib, whereas arterial occlusive and metabolic events are more prominent with nilotinib and ponatinib, and imatinib generally shows a more favorable cardiometabolic profile, a pattern that has also been observed in other clinical and FAERS-based evaluations of BCR-ABL1 TKIs (10, 19, 20). However, our analysis provides a real-world perspective that may capture AEs not fully represented in controlled clinical trials.

Identifying hepatotoxicity as a major signal is consistent with preclinical and clinical studies demonstrating the potential of dasatinib for liver injury (50, 51). The relative signal strength from our analysis (ROR = 29.17) provide a valuable context for clinical decision-making and patient counseling. Similarly, cardiovascular signals align with emerging concerns regarding tyrosine kinase inhibitor-associated vascular toxicities, although dasatinib appears to have a more favorable cardiovascular profile compared to other agents of this class (52).

The novel signals identified in our analysis, such as alveolar proteinosis and cytomegalovirus-related complications, extend beyond the currently recognized adverse effects and may represent important areas for future clinical investigation. These findings highlight the value of large-scale pharmacovigilance studies in identifying rare but potentially significant AEs that might not be captured in clinical trials.

### Limitations and methodological considerations

Several limitations inherent to FAERS-based pharmacovigilance studies must be addressed. The spontaneous reporting nature of FAERS introduces a potential reporting bias, with serious or unexpected events being more likely to be reported than mild or well-known effects. Additionally, the database lacks comprehensive denominator data, preventing the calculation of true incidence rates and limiting risk quantification to disproportionate measures. Information on treatment indication, disease phase, comorbidities, dosing, treatment duration, and prior TKIs is also limited, so residual confounding by disease severity and treatment selection is unavoidable; many hematologic and transplantation-related PTs may therefore reflect the underlying course of advanced leukemia rather than *de novo* dasatinib toxicity. Despite being addressed through standard deduplication procedures, duplicate reporting may still influence signal strength. Furthermore, the retrospective nature of the analysis and reliance on medical terminology coding may have introduced classification errors or missed relevant events coded using different terms, and all signals should be regarded as hypothesis-generating rather than proof of causality.

## Conclusion

This FAERS-based pharmacovigilance analysis confirmed the well-known risks of dasatinib, especially respiratory complications, such as pleural effusion, while identifying important hepatotoxicity, cardiovascular, and novel adverse event signals. The early failure pattern of AEs underscores the need for vigilant monitoring, both during treatment initiation and over the long-term. Subgroup differences suggest that surveillance should be tailored to patient demographics. Mechanistic insights highlight the off-target effects of dasatinib in multiple organ systems. These findings support optimized clinical management and further investigation of rare and emerging toxicities to improve patient safety.

## Data Availability

The original contributions presented in this study are included in this article/Supplementary material, further inquiries can be directed to the corresponding authors.
