# Supplementary material for: Toward the future management of patients with CML and Ph + ALL: real-world safety insights from dasatinib pharmacovigilance
Source: Front Med (Lausanne). 2026 Jan 6;12:1709089. doi: 10.3389/fmed.2025.1709089 (PMC12815812; doi:10.3389/fmed.2025.1709089)
Supplement: Supplementary file 2 [file Supplementary_file_2.docx]

**Supplementary Table 1** Two-by-two contingency table for disproportionality analyses.

|  | Target AEs | Other AEs | Total |
| --- | --- | --- | --- |
| Target drugs | a | b | a+b |
| Other drugs | c | d | c+d |
| Total | a+c | b+d | a+b+c+d |

Abbreviation: AEs, adverse events; a, number of reports containing both the target drug and target adverse drug reaction; b, number of reports containing other adverse drug reaction of the target drug; c, number of reports containing the target adverse drug reaction of other drugs; d, number of reports containing other drugs and other adverse drug reactions.

**Supplementary Table 2** Four major algorithms used for signal detection.

| Algorithms | Calculation Formula | Threshold |
| --- | --- | --- |
| ROR | $\begin{matrix} ROR=\frac{(a/c)}{(b/d)}=\frac{ad}{bc} \\ SE(InROR)=\sqrt{\left(\frac{1}{a}+\frac{1}{b}+\frac{1}{c}+\frac{1}{d}\right)} \\ 95\%CI=eIn(ROR)\pm1.96\sqrt{\left(\frac{1}{a}+\frac{1}{b}+\frac{1}{c}+\frac{1}{d}\right)} \end{matrix}$ | a≥3 with a lower 95% CI > 1 |
| PRR | $\begin{matrix} PRR=\frac{\text{a}/\left(a+b\right)}{c/(c+d)} \\ SE(InPRR)=\sqrt{\frac{1}{a}-\frac{1}{a+b}+\frac{1}{c}-\frac{1}{c+d}} \\ 95\%CI=eIn(PRR)\pm1.96\sqrt{(\frac{1}{a}-\frac{1}{a+b}+\frac{1}{c}-\frac{1}{c+d})} \end{matrix}$ | a≥3 with a lower 95% CI > 1 |
| BCPNN | α1=β1=1; α=β=2; γ11=1 | (-): E(IC)≤0  (+):0< E(IC) ≤1.5:  (++):1.5< E(IC) ≤3:  (+++):E(IC)>3 |
| MGPS |  | EBGM05>2,a>0 |

Abbreviation: a number of reports containing both the target drug and target adverse drug reaction; b, number of reports containing other adverse drug reaction of the target drug; c, number of reports containing the target adverse drug reaction of other drugs; d, number of reports containing other drugs and other adverse drug reactions. 95%CI, 95% confidence interval; N, the number of reports; χ2, chi-squared; IC, information component; IC025, the lower limit of 95% CI of the IC; E(IC), the IC expectations; V(IC), the variance of IC; EBGM, empirical Bayesian geometric mean; EBGM05, the lower limit of 95% CI of EBGM.

**Supplementary Table 3** Distribution of AEs according to PT in female patients.

| PT | Case number | ROR(95%Cl) | PRR(XX) | EBGM(EBGM05) | IC(IC025) |
| --- | --- | --- | --- | --- | --- |
| Blast cell proliferation | 3 | 813.98(182.14-3637.63) | 813.65(1391.4) | 465.37(104.13) | 8.86(0.22) |
| Chylothorax | 15 | 214.55(123.26-373.46) | 214.12(2657.39) | 178.99(102.83) | 7.48(3.1) |
| Philadelphia chromosome positive | 9 | 112.36(56.55-223.28) | 112.23(899.13) | 101.8(51.23) | 6.67(2.24) |
| Aspiration pleural cavity | 8 | 104.68(50.65-216.34) | 104.57(748.47) | 95.46(46.19) | 6.58(2.04) |
| Exposure via body fluid | 20 | 76.88(48.82-121.06) | 76.67(1395.1) | 71.67(45.52) | 6.16(3.38) |
| Chronic myeloid leukaemia recurrent | 4 | 73.59(26.72-202.64) | 73.55(268.08) | 68.94(25.04) | 6.11(0.9) |
| Cytogenetic analysis abnormal | 12 | 70.1(39.09-125.74) | 69.99(766.63) | 65.81(36.69) | 6.04(2.63) |
| Lymphoid tissue hyperplasia | 4 | 60.3(22.03-165.09) | 60.27(220.88) | 57.15(20.88) | 5.84(0.89) |
| Chronic myeloid leukaemia transformation | 3 | 47.19(14.85-149.95) | 47.17(129.92) | 45.24(14.24) | 5.5(0.42) |
| Neutrophilic dermatosis | 3 | 42.84(13.51-135.83) | 42.82(117.89) | 41.24(13.01) | 5.37(0.42) |
| Pleural effusion | 325 | 33.81(30.21-37.85) | 32.36(9605) | 31.45(28.1) | 4.98(4.68) |
| Bone marrow transplant | 5 | 30.32(12.47-73.77) | 30.3(137.84) | 29.51(12.13) | 4.88(1.16) |
| Peripheral artery stenosis | 3 | 27.36(8.7-86.07) | 27.35(74.29) | 26.7(8.49) | 4.74(0.38) |
| Prescribed underdose | 50 | 25.84(19.5-34.24) | 25.67(1158.42) | 25.1(18.94) | 4.65(3.68) |
| Gene mutation | 4 | 24.26(9-65.35) | 24.24(87.19) | 23.73(8.81) | 4.57(0.79) |
| Acute lymphocytic leukaemia recurrent | 8 | 24.07(11.94-48.51) | 24.04(172.84) | 23.54(11.68) | 4.56(1.77) |
| Lymphocytosis | 9 | 22.95(11.85-44.42) | 22.92(184.78) | 22.47(11.6) | 4.49(1.91) |
| Transplant | 4 | 22.27(8.27-59.94) | 22.25(79.56) | 21.83(8.11) | 4.45(0.77) |
| Cytomegalovirus enterocolitis | 3 | 21.71(6.92-68.08) | 21.7(58.07) | 21.29(6.79) | 4.41(0.35) |
| Blast crisis in myelogenous leukaemia | 3 | 20.48(6.53-64.18) | 20.47(54.53) | 20.11(6.42) | 4.33(0.34) |
| Stem cell transplant | 5 | 19.52(8.06-47.29) | 19.51(86.27) | 19.18(7.92) | 4.26(1.06) |
| Hydrothorax | 3 | 18.6(5.94-58.26) | 18.6(49.11) | 18.3(5.84) | 4.19(0.32) |
| Demyelinating polyneuropathy | 3 | 17.99(5.75-56.31) | 17.98(47.33) | 17.7(5.66) | 4.15(0.31) |
| Enterocolitis haemorrhagic | 6 | 16.83(7.51-37.71) | 16.82(87.92) | 16.58(7.4) | 4.05(1.26) |
| Intermittent claudication | 4 | 16.76(6.24-45.01) | 16.75(58.36) | 16.52(6.15) | 4.05(0.7) |
| Skin depigmentation | 3 | 16.7(5.34-52.23) | 16.69(43.58) | 16.45(5.26) | 4.04(0.3) |
| Cytomegalovirus colitis | 5 | 16.45(6.8-39.79) | 16.44(71.41) | 16.21(6.7) | 4.02(1.01) |
| Microangiopathy | 3 | 16.36(5.23-51.17) | 16.35(42.61) | 16.13(5.16) | 4.01(0.3) |
| Leukaemia recurrent | 3 | 16.04(5.13-50.16) | 16.03(41.67) | 15.81(5.06) | 3.98(0.29) |
| Pericardial effusion | 56 | 15.53(11.91-20.23) | 15.42(744.7) | 15.21(11.67) | 3.93(3.22) |
| Pulmonary oedema | 74 | 13.65(10.84-17.18) | 13.52(848.01) | 13.37(10.62) | 3.74(3.18) |
| Pleurisy | 14 | 13.2(7.79-22.36) | 13.17(155.61) | 13.03(7.69) | 3.7(2.1) |
| Fluid retention | 53 | 11.69(8.91-15.34) | 11.61(508.95) | 11.5(8.77) | 3.52(2.87) |
| Graft versus host disease | 8 | 11.31(5.63-22.71) | 11.3(74.35) | 11.19(5.58) | 3.48(1.42) |
| Peripheral arterial occlusive disease | 6 | 10.16(4.55-22.71) | 10.15(49.06) | 10.07(4.51) | 3.33(1.04) |
| Chronic myeloid leukaemia | 6 | 9.77(4.37-21.82) | 9.76(46.75) | 9.68(4.33) | 3.28(1.01) |
| Therapy change | 12 | 9.71(5.5-17.16) | 9.7(92.83) | 9.62(5.45) | 3.27(1.73) |
| Adverse event | 63 | 9.48(7.39-12.16) | 9.4(469.5) | 9.33(7.27) | 3.22(2.68) |
| Pulmonary hypertension | 35 | 9.35(6.7-13.06) | 9.31(257.65) | 9.24(6.62) | 3.21(2.43) |
| Drug resistance | 19 | 9.13(5.81-14.35) | 9.11(136.12) | 9.04(5.76) | 3.18(2.04) |
| Acute lymphocytic leukaemia | 4 | 8.72(3.26-23.33) | 8.71(27.1) | 8.65(3.23) | 3.11(0.48) |
| Drug tolerance decreased | 3 | 8.64(2.77-26.9) | 8.63(20.09) | 8.57(2.75) | 3.1(0.12) |
| Normal newborn | 9 | 8.46(4.39-16.3) | 8.45(58.63) | 8.39(4.35) | 3.07(1.35) |
| Pulmonary arterial hypertension | 20 | 8.36(5.38-12.99) | 8.34(128.29) | 8.29(5.33) | 3.05(1.99) |
| Skin toxicity | 8 | 8.27(4.13-16.59) | 8.27(50.71) | 8.21(4.09) | 3.04(1.22) |
| Periorbital oedema | 7 | 8.16(3.88-17.18) | 8.16(43.63) | 8.1(3.85) | 3.02(1.08) |
| Splenomegaly | 11 | 8.04(4.44-14.55) | 8.03(67.16) | 7.97(4.4) | 3(1.5) |
| Cytomegalovirus infection reactivation | 3 | 7.81(2.51-24.31) | 7.8(17.67) | 7.76(2.49) | 2.96(0.08) |
| Lung consolidation | 3 | 7.77(2.5-24.2) | 7.77(17.56) | 7.72(2.48) | 2.95(0.08) |
| Hair texture abnormal | 4 | 7.71(2.88-20.62) | 7.71(23.19) | 7.66(2.86) | 2.94(0.42) |
| Acute febrile neutrophilic dermatosis | 3 | 7.42(2.38-23.09) | 7.41(16.53) | 7.37(2.37) | 2.88(0.06) |
| Hospitalisation | 95 | 7.38(6.03-9.04) | 7.3(513.93) | 7.26(5.92) | 2.86(2.47) |
| Haematotoxicity | 10 | 7.37(3.95-13.73) | 7.36(54.6) | 7.32(3.93) | 2.87(1.34) |
| Vitreous haemorrhage | 3 | 6.71(2.16-20.89) | 6.71(14.49) | 6.68(2.14) | 2.74(0.01) |
| Blood disorder | 5 | 6.12(2.54-14.74) | 6.12(21.28) | 6.09(2.53) | 2.61(0.54) |
| Iron deficiency | 4 | 5.97(2.23-15.96) | 5.97(16.46) | 5.94(2.22) | 2.57(0.28) |
| Pulmonary congestion | 9 | 5.76(2.99-11.09) | 5.75(35.17) | 5.73(2.97) | 2.52(1.04) |
| Nephrotic syndrome | 6 | 5.54(2.48-12.36) | 5.54(22.18) | 5.51(2.47) | 2.46(0.65) |
| Cytopenia | 10 | 5.36(2.88-9.97) | 5.35(35.2) | 5.33(2.86) | 2.41(1.06) |
| Transfusion | 6 | 5.21(2.33-11.62) | 5.2(20.28) | 5.18(2.32) | 2.37(0.6) |
| Tumour lysis syndrome | 6 | 5.1(2.29-11.39) | 5.1(19.69) | 5.08(2.28) | 2.35(0.59) |
| Acne | 16 | 5.04(3.08-8.24) | 5.03(51.49) | 5.01(3.07) | 2.33(1.32) |
| Malignant neoplasm progression | 74 | 4.96(3.94-6.24) | 4.92(230.55) | 4.9(3.9) | 2.29(1.88) |
| Bone pain | 39 | 4.95(3.61-6.79) | 4.93(121.87) | 4.92(3.59) | 2.3(1.7) |
| Therapy cessation | 26 | 4.92(3.34-7.23) | 4.9(80.43) | 4.88(3.32) | 2.29(1.54) |
| Large intestine polyp | 5 | 4.9(2.04-11.81) | 4.9(15.45) | 4.88(2.03) | 2.29(0.39) |
| Skin disorder | 18 | 4.86(3.06-7.73) | 4.85(54.8) | 4.83(3.04) | 2.27(1.34) |
| Full blood count decreased | 13 | 4.47(2.59-7.71) | 4.46(34.79) | 4.45(2.58) | 2.15(1.06) |
| Intentional product use issue | 29 | 4.41(3.06-6.35) | 4.39(75.79) | 4.38(3.04) | 2.13(1.45) |
| Lung infiltration | 8 | 4.3(2.15-8.61) | 4.3(20.16) | 4.28(2.14) | 2.1(0.68) |
| Ulcer | 7 | 4.29(2.04-9.02) | 4.29(17.58) | 4.28(2.03) | 2.1(0.58) |
| Alopecia | 76 | 3.57(2.85-4.48) | 3.55(139.05) | 3.54(2.82) | 1.82(1.45) |
| Oedema | 29 | 3.43(2.38-4.95) | 3.42(49.69) | 3.42(2.37) | 1.77(1.13) |
| Cardiac disorder | 20 | 3.18(2.05-4.93) | 3.17(29.69) | 3.17(2.04) | 1.66(0.89) |
| Fatigue | 196 | 2.61(2.26-3) | 2.56(188.51) | 2.56(2.22) | 1.36(1.14) |
| Rash | 153 | 2.45(2.09-2.88) | 2.42(128.6) | 2.42(2.06) | 1.27(1.03) |
| Diarrhoea | 184 | 2.45(2.12-2.84) | 2.42(153.87) | 2.41(2.08) | 1.27(1.04) |
| Headache | 169 | 2.44(2.09-2.84) | 2.41(139.79) | 2.4(2.06) | 1.26(1.03) |

Abbreviations: PT, Preferred Term; ROR, Reporting Odds Ratio; PRR, Proportional Reporting Ratio; EBGM, Empirical Bayes Geometric Mean; EBGM05, lower 5% one-sided confidence limit of EBGM; IC, Information Component; IC025, lower end of the 95% credibility interval of IC.

**Supplementary Table 4** Distribution of AEs according to PT in male patients.

| PT | Case number | ROR(95%Cl) | PRR(XX) | EBGM(EBGM05) | IC(IC025) |
| --- | --- | --- | --- | --- | --- |
| Blast cell proliferation | 7 | 494.83(191.77-1276.83) | 494.36(2106.23) | 302.5(117.23) | 8.24(1.75) |
| Allogenic bone marrow transplantation therapy | 3 | 194.29(54.82-688.66) | 194.21(461.33) | 155.57(43.89) | 7.28(0.36) |
| Chylothorax | 21 | 144.78(90.82-230.8) | 144.37(2521.43) | 121.9(76.47) | 6.93(3.56) |
| Primary effusion lymphoma | 3 | 122.71(36.3-414.77) | 122.66(312.64) | 106.07(31.38) | 6.73(0.39) |
| Philadelphia chromosome positive | 17 | 116.11(69.72-193.38) | 115.85(1684.4) | 100.94(60.61) | 6.66(3.22) |
| Lymphoid tissue hyperplasia | 7 | 115.81(52.33-256.3) | 115.7(692.8) | 100.83(45.56) | 6.66(1.81) |
| Aspiration pleural cavity | 11 | 93.02(49.76-173.91) | 92.88(893.09) | 83.07(44.43) | 6.38(2.52) |
| Chronic myeloid leukaemia transformation | 5 | 56.33(22.72-139.67) | 56.29(253.21) | 52.56(21.2) | 5.72(1.23) |
| Chronic myeloid leukaemia recurrent | 4 | 53.6(19.46-147.69) | 53.58(193.07) | 50.18(18.21) | 5.65(0.87) |
| Acquired gene mutation | 4 | 41.45(15.16-113.37) | 41.43(149.84) | 39.39(14.4) | 5.3(0.85) |
| Blast crisis in myelogenous leukaemia | 9 | 36.84(18.87-71.93) | 36.8(299.26) | 35.18(18.02) | 5.14(2.06) |
| Cytogenetic analysis abnormal | 8 | 35.96(17.7-73.09) | 35.92(259.62) | 34.38(16.92) | 5.1(1.88) |
| Pleural effusion | 406 | 32.24(29.11-35.69) | 30.51(11173.26) | 29.4(26.55) | 4.88(4.63) |
| Bone marrow transplant | 8 | 31.9(15.73-64.73) | 31.87(229.8) | 30.65(15.11) | 4.94(1.85) |
| Gene mutation identification test positive | 3 | 29.14(9.2-92.3) | 29.13(78.55) | 28.12(8.88) | 4.81(0.38) |
| Polyserositis | 3 | 28.43(8.98-90.01) | 28.42(76.57) | 27.45(8.67) | 4.78(0.37) |
| Enterocolitis haemorrhagic | 11 | 26.91(14.75-49.11) | 26.87(264.86) | 26.01(14.25) | 4.7(2.23) |
| Prescribed underdose | 61 | 26.72(20.68-34.52) | 26.5(1448.09) | 25.66(19.86) | 4.68(3.82) |
| Minimal residual disease | 3 | 26.2(8.29-82.79) | 26.19(70.3) | 25.36(8.03) | 4.66(0.36) |
| Alveolar proteinosis | 3 | 25.91(8.2-81.86) | 25.9(69.49) | 25.09(7.94) | 4.65(0.36) |
| Platelet transfusion | 4 | 23.92(8.84-64.7) | 23.9(85.16) | 23.22(8.58) | 4.54(0.78) |
| Anorectal polyp | 4 | 23.73(8.77-64.2) | 23.72(84.47) | 23.05(8.52) | 4.53(0.78) |
| Hyperplasia | 4 | 23.55(8.71-63.71) | 23.54(83.8) | 22.88(8.46) | 4.52(0.77) |
| Gene mutation | 5 | 19.73(8.12-47.94) | 19.72(86.64) | 19.25(7.92) | 4.27(1.05) |
| Fluid retention | 67 | 17.94(14.07-22.88) | 17.79(1038.38) | 17.41(13.65) | 4.12(3.45) |
| Pleurisy | 19 | 17.01(10.79-26.81) | 16.97(279.41) | 16.62(10.55) | 4.06(2.57) |
| Acute promyelocytic leukaemia | 3 | 16.19(5.16-50.81) | 16.18(41.87) | 15.87(5.06) | 3.99(0.29) |
| Right ventricular dysfunction | 3 | 15.65(4.99-49.08) | 15.64(40.31) | 15.35(4.89) | 3.94(0.28) |
| Drug tolerance decreased | 3 | 15.04(4.8-47.16) | 15.04(38.56) | 14.77(4.71) | 3.88(0.27) |
| Ejection fraction abnormal | 3 | 14.22(4.54-44.55) | 14.21(36.18) | 13.97(4.46) | 3.8(0.26) |
| Therapy change | 18 | 14.04(8.81-22.39) | 14.01(213.67) | 13.78(8.64) | 3.78(2.37) |
| Transplant | 4 | 13.76(5.12-36.98) | 13.75(46.47) | 13.53(5.03) | 3.76(0.64) |
| Pleural fibrosis | 3 | 13.32(4.25-41.72) | 13.32(33.6) | 13.11(4.19) | 3.71(0.24) |
| Chronic myeloid leukaemia | 14 | 13.05(7.69-22.14) | 13.03(152.89) | 12.83(7.56) | 3.68(2.09) |
| Pulmonary hypertension | 40 | 12.84(9.39-17.57) | 12.78(427.38) | 12.59(9.2) | 3.65(2.84) |
| Adverse event | 70 | 12.31(9.71-15.61) | 12.2(709.42) | 12.03(9.49) | 3.59(3.03) |
| Pericardial effusion | 53 | 12.19(9.28-16) | 12.11(532.04) | 11.94(9.09) | 3.58(2.91) |
| Acute lymphocytic leukaemia recurrent | 7 | 11.46(5.43-24.17) | 11.45(65.79) | 11.3(5.35) | 3.5(1.27) |
| Blast cell count increased | 3 | 11(3.52-34.38) | 10.99(26.87) | 10.85(3.47) | 3.44(0.19) |
| Pulmonary oedema | 75 | 10.53(8.37-13.24) | 10.43(631.62) | 10.31(8.2) | 3.37(2.86) |
| Sunburn | 6 | 10.37(4.63-23.2) | 10.36(50.06) | 10.23(4.57) | 3.36(1.04) |
| Lymphocytosis | 6 | 9.92(4.43-22.21) | 9.92(47.51) | 9.8(4.38) | 3.29(1.02) |
| Transfusion | 11 | 9.23(5.09-16.73) | 9.22(79.66) | 9.12(5.03) | 3.19(1.6) |
| Bone marrow disorder | 4 | 9.12(3.4-24.44) | 9.11(28.56) | 9.02(3.36) | 3.17(0.49) |
| Sinus headache | 3 | 9.07(2.91-28.32) | 9.07(21.29) | 8.98(2.87) | 3.17(0.13) |
| Cytomegalovirus colitis | 4 | 9.06(3.38-24.29) | 9.06(28.35) | 8.97(3.35) | 3.16(0.49) |
| Acne | 23 | 8.61(5.71-13) | 8.59(152.62) | 8.51(5.64) | 3.09(2.1) |
| Red blood cell transfusion | 3 | 8.51(2.73-26.55) | 8.51(19.66) | 8.42(2.7) | 3.07(0.11) |
| Haemothorax | 7 | 7.96(3.78-16.76) | 7.95(42.11) | 7.88(3.74) | 2.98(1.06) |
| Internal haemorrhage | 6 | 7.88(3.52-17.62) | 7.87(35.65) | 7.8(3.49) | 2.96(0.89) |
| Eye haemorrhage | 8 | 7.58(3.78-15.21) | 7.57(45.18) | 7.51(3.74) | 2.91(1.15) |
| Periorbital oedema | 6 | 7.5(3.35-16.76) | 7.49(33.44) | 7.43(3.32) | 2.89(0.86) |
| Pulmonary arterial hypertension | 11 | 7.1(3.92-12.86) | 7.09(57.05) | 7.04(3.88) | 2.81(1.39) |
| Stem cell transplant | 3 | 6.96(2.23-21.69) | 6.96(15.17) | 6.9(2.21) | 2.79(0.03) |
| Magnetic resonance imaging abnormal | 3 | 6.92(2.22-21.56) | 6.92(15.05) | 6.86(2.2) | 2.78(0.02) |
| Graft versus host disease in gastrointestinal tract | 3 | 6.74(2.16-21) | 6.74(14.53) | 6.69(2.15) | 2.74(0.01) |
| Product administered to patient of inappropriate age | 7 | 6.29(2.99-13.24) | 6.29(30.88) | 6.24(2.97) | 2.64(0.89) |
| Enterocolitis | 10 | 6.25(3.35-11.64) | 6.24(43.66) | 6.2(3.33) | 2.63(1.2) |
| Therapy cessation | 32 | 5.89(4.16-8.35) | 5.87(128.37) | 5.83(4.12) | 2.54(1.84) |
| Hospitalisation | 92 | 5.64(4.59-6.93) | 5.58(344.02) | 5.55(4.51) | 2.47(2.1) |
| Insurance issue | 4 | 5.51(2.06-14.74) | 5.51(14.66) | 5.48(2.05) | 2.45(0.23) |
| Eye swelling | 14 | 5.49(3.24-9.29) | 5.48(50.96) | 5.45(3.22) | 2.45(1.32) |
| Malignant neoplasm progression | 115 | 5.47(4.54-6.58) | 5.4(410.29) | 5.37(4.46) | 2.42(2.1) |
| Bone pain | 22 | 5.27(3.46-8.02) | 5.26(75.34) | 5.23(3.43) | 2.39(1.54) |
| Full blood count decreased | 16 | 5.17(3.16-8.45) | 5.16(53.29) | 5.13(3.14) | 2.36(1.34) |
| Graft versus host disease | 8 | 5.16(2.57-10.34) | 5.15(26.61) | 5.13(2.56) | 2.36(0.85) |
| Drug resistance | 18 | 4.9(3.08-7.8) | 4.89(55.43) | 4.87(3.06) | 2.28(1.35) |
| Leukaemia | 6 | 4.88(2.19-10.9) | 4.88(18.4) | 4.86(2.18) | 2.28(0.55) |
| Drug intolerance | 47 | 4.78(3.59-6.37) | 4.76(138.75) | 4.73(3.55) | 2.24(1.72) |
| Respiratory tract congestion | 6 | 4.59(2.05-10.24) | 4.58(16.71) | 4.56(2.04) | 2.19(0.5) |
| Eyelid oedema | 7 | 4.38(2.08-9.21) | 4.38(18.15) | 4.36(2.07) | 2.12(0.59) |
| Skin disorder | 12 | 3.68(2.09-6.49) | 3.67(23.26) | 3.66(2.08) | 1.87(0.8) |
| Headache | 149 | 3.45(2.93-4.06) | 3.4(253.01) | 3.39(2.88) | 1.76(1.5) |
| Intentional product use issue | 27 | 3.35(2.3-4.9) | 3.35(44.25) | 3.34(2.28) | 1.74(1.07) |
| Oedema | 26 | 3.16(2.15-4.64) | 3.15(37.99) | 3.14(2.13) | 1.65(0.98) |
| Fatigue | 189 | 2.86(2.48-3.31) | 2.82(222.46) | 2.81(2.43) | 1.49(1.26) |

Abbreviations: PT, Preferred Term; ROR, Reporting Odds Ratio; PRR, Proportional Reporting Ratio; EBGM, Empirical Bayes Geometric Mean; EBGM05, lower 5% one-sided confidence limit of EBGM; IC, Information Component; IC025, lower end of the 95% credibility interval of IC.

**Supplementary Table 5** Distribution of AEs according to PT aged <18 years.

| PT | Case number | ROR(95%Cl) | PRR(XX) | EBGM(EBGM05) | IC(IC025) |
| --- | --- | --- | --- | --- | --- |
| Transplant | 4 | 222.63(76.77-645.66) | 221.19(746.92) | 188.57(65.02) | 7.56(0.88) |
| Chylothorax | 3 | 174.28(52.03-583.8) | 173.44(452.62) | 152.74(45.6) | 7.25(0.42) |
| Enterocolitis haemorrhagic | 5 | 105.1(42.08-262.45) | 104.25(472.61) | 96.43(38.61) | 6.59(1.28) |
| Bone marrow disorder | 3 | 67.26(21.01-215.36) | 66.94(185.14) | 63.64(19.88) | 5.99(0.44) |
| Prescribed underdose | 9 | 54.52(27.85-106.73) | 53.74(447.07) | 51.6(26.36) | 5.69(2.15) |
| Pleural effusion | 41 | 51.94(37.63-71.71) | 48.55(1841.93) | 46.8(33.9) | 5.55(4.01) |
| Full blood count decreased | 3 | 51.81(16.29-164.78) | 51.56(142.96) | 49.59(15.59) | 5.63(0.43) |
| Pleurisy | 3 | 47.33(14.91-150.24) | 47.11(130.56) | 45.46(14.32) | 5.51(0.43) |
| Full blood count abnormal | 3 | 45.64(14.39-144.77) | 45.42(125.86) | 43.89(13.84) | 5.46(0.42) |
| Therapy change | 4 | 44.91(16.52-122.11) | 44.63(164.84) | 43.15(15.87) | 5.43(0.87) |
| Enterocolitis | 6 | 34.24(15.16-77.33) | 33.92(186.75) | 33.06(14.64) | 5.05(1.45) |
| Fluid retention | 4 | 32.4(11.97-87.68) | 32.2(117.96) | 31.43(11.61) | 4.97(0.83) |
| Colon cancer | 4 | 21.69(8.05-58.46) | 21.56(77.13) | 21.21(7.87) | 4.41(0.76) |
| Fracture | 5 | 16.91(6.97-41) | 16.78(73.26) | 16.57(6.83) | 4.05(1.01) |
| Nephrotic syndrome | 4 | 16.25(6.04-43.7) | 16.15(56.16) | 15.96(5.93) | 4(0.69) |
| Encephalitis | 3 | 12.57(4.02-39.29) | 12.51(31.47) | 12.4(3.97) | 3.63(0.23) |
| Oedema | 7 | 12.38(5.85-26.16) | 12.25(71.67) | 12.14(5.74) | 3.6(1.31) |
| Clostridium difficile infection | 3 | 11.44(3.66-35.75) | 11.39(28.19) | 11.3(3.62) | 3.5(0.2) |
| Intentional product use issue | 7 | 9.75(4.61-20.59) | 9.65(53.9) | 9.58(4.54) | 3.26(1.18) |
| Hospitalisation | 9 | 8.48(4.38-16.41) | 8.37(58.1) | 8.32(4.3) | 3.06(1.34) |
| Malignant neoplasm progression | 8 | 7.76(3.86-15.62) | 7.67(46.23) | 7.63(3.79) | 2.93(1.16) |
| Product administered to patient of inappropriate age | 7 | 6.65(3.15-14.03) | 6.59(33.04) | 6.56(3.11) | 2.71(0.92) |
| Gastrointestinal haemorrhage | 5 | 6.07(2.51-14.68) | 6.03(20.93) | 6.01(2.49) | 2.59(0.52) |
| Death | 15 | 5.15(3.08-8.61) | 5.05(48.8) | 5.04(3.01) | 2.33(1.27) |
| PT | Case number | ROR(95%Cl) | PRR(XX) | EBGM(EBGM05) | IC(IC025) |
| Transplant | 4 | 222.63(76.77-645.66) | 221.19(746.92) | 188.57(65.02) | 7.56(0.88) |
| Chylothorax | 3 | 174.28(52.03-583.8) | 173.44(452.62) | 152.74(45.6) | 7.25(0.42) |

Abbreviations: PT, Preferred Term; ROR, Reporting Odds Ratio; PRR, Proportional Reporting Ratio; EBGM, Empirical Bayes Geometric Mean; EBGM05, lower 5% one-sided confidence limit of EBGM; IC, Information Component; IC025, lower end of the 95% credibility interval of IC.

**Supplementary Table 6** Distribution of AEs according to PT aged 18-65.

| PT | Case number | ROR(95%Cl) | PRR(XX) | EBGM(EBGM05) | IC(IC025) |
| --- | --- | --- | --- | --- | --- |
| Blast cell proliferation | 6 | 945.53(304.87-2932.49) | 944.7(2828.11) | 472.85(152.46) | 8.89(1.43) |
| Chylothorax | 19 | 243.23(146.85-402.89) | 242.56(3636.95) | 193.21(116.64) | 7.59(3.47) |
| Philadelphia chromosome positive | 12 | 153.46(83.35-282.56) | 153.19(1561.26) | 131.96(71.67) | 7.04(2.71) |
| Aspiration pleural cavity | 8 | 109.66(52.72-228.1) | 109.53(770.94) | 98.25(47.24) | 6.62(2.04) |
| Chronic myeloid leukaemia transformation | 5 | 78.78(31.63-196.24) | 78.73(354.18) | 72.75(29.2) | 6.18(1.26) |
| Lymphoid tissue hyperplasia | 6 | 70.91(30.92-162.62) | 70.85(384.38) | 65.98(28.77) | 6.04(1.55) |
| Cytogenetic analysis abnormal | 9 | 55.64(28.4-109.03) | 55.57(455.52) | 52.54(26.81) | 5.72(2.15) |
| Bone marrow transplant | 9 | 50.37(25.75-98.54) | 50.31(412.98) | 47.82(24.45) | 5.58(2.13) |
| Blast crisis in myelogenous leukaemia | 7 | 42.16(19.77-89.93) | 42.12(269.02) | 40.37(18.92) | 5.34(1.72) |
| Neutrophilic dermatosis | 3 | 38.84(12.24-123.26) | 38.82(106.18) | 37.33(11.76) | 5.22(0.41) |
| Pleural effusion | 283 | 32.46(28.76-36.63) | 31.15(8005.64) | 30.19(26.75) | 4.92(4.6) |
| Acquired gene mutation | 4 | 32.32(11.93-87.57) | 32.3(117.31) | 31.26(11.54) | 4.97(0.83) |
| Transplant | 5 | 27.97(11.49-68.09) | 27.95(126.2) | 27.18(11.16) | 4.76(1.14) |
| Enterocolitis haemorrhagic | 9 | 23.32(12.03-45.21) | 23.29(187.42) | 22.76(11.74) | 4.51(1.91) |
| Gene mutation | 5 | 23.06(9.49-56.01) | 23.04(102.92) | 22.52(9.27) | 4.49(1.1) |
| Right ventricular dysfunction | 4 | 21.24(7.88-57.24) | 21.23(75.41) | 20.78(7.71) | 4.38(0.76) |
| Prescribed underdose | 30 | 19.63(13.66-28.2) | 19.55(517.32) | 19.17(13.34) | 4.26(3.07) |
| Hyperplasia | 3 | 17.72(5.65-55.54) | 17.71(46.44) | 17.41(5.55) | 4.12(0.31) |
| Cytomegalovirus enterocolitis | 3 | 17.72(5.65-55.54) | 17.71(46.44) | 17.41(5.55) | 4.12(0.31) |
| Chronic myeloid leukaemia | 12 | 17.28(9.76-30.61) | 17.25(180.48) | 16.96(9.58) | 4.08(2.12) |
| Lymphocytosis | 8 | 16.96(8.43-34.15) | 16.95(117.93) | 16.66(8.28) | 4.06(1.63) |
| Fluid retention | 54 | 15.42(11.78-20.2) | 15.31(711.14) | 15.08(11.51) | 3.91(3.19) |
| Haemothorax | 6 | 15.21(6.79-34.08) | 15.2(78.32) | 14.97(6.68) | 3.9(1.22) |
| Pleurisy | 16 | 14.13(8.62-23.16) | 14.1(191.9) | 13.91(8.48) | 3.8(2.28) |
| Therapy change | 18 | 13.87(8.7-22.1) | 13.84(211.3) | 13.65(8.57) | 3.77(2.37) |
| Pericardial effusion | 49 | 13.61(10.25-18.06) | 13.52(560.17) | 13.34(10.05) | 3.74(3.01) |
| Pulmonary hypertension | 35 | 12.97(9.29-18.12) | 12.91(379.56) | 12.75(9.13) | 3.67(2.78) |
| Cytomegalovirus colitis | 5 | 12.77(5.28-30.88) | 12.77(53.5) | 12.61(5.22) | 3.66(0.91) |
| Full blood count decreased | 16 | 12.09(7.38-19.81) | 12.06(160.32) | 11.92(7.28) | 3.58(2.15) |
| Stem cell transplant | 5 | 11.64(4.82-28.13) | 11.63(48.01) | 11.5(4.76) | 3.52(0.88) |
| Demyelinating polyneuropathy | 3 | 11.57(3.71-36.14) | 11.57(28.61) | 11.44(3.66) | 3.52(0.21) |
| Pulmonary arterial hypertension | 19 | 10.17(6.47-16) | 10.15(155.04) | 10.05(6.39) | 3.33(2.14) |
| Drug tolerance decreased | 3 | 9.68(3.1-30.18) | 9.67(23.09) | 9.58(3.07) | 3.26(0.15) |
| Sunburn | 5 | 9.21(3.82-22.24) | 9.21(36.23) | 9.13(3.78) | 3.19(0.77) |
| Eye haemorrhage | 7 | 9.02(4.28-18.99) | 9.01(49.38) | 8.93(4.24) | 3.16(1.14) |
| Internal haemorrhage | 3 | 8.86(2.84-27.62) | 8.86(20.72) | 8.78(2.82) | 3.13(0.12) |
| Graft versus host disease in gastrointestinal tract | 3 | 8.75(2.81-27.28) | 8.75(20.4) | 8.68(2.78) | 3.12(0.12) |
| Pulmonary oedema | 44 | 8.57(6.36-11.54) | 8.52(289.64) | 8.45(6.28) | 3.08(2.42) |
| Acne | 26 | 8.56(5.81-12.6) | 8.53(171.32) | 8.46(5.75) | 3.08(2.17) |
| Graft versus host disease | 11 | 8.45(4.66-15.3) | 8.43(71.46) | 8.37(4.62) | 3.07(1.54) |
| Gingival disorder | 3 | 8.36(2.68-26.07) | 8.36(19.27) | 8.3(2.66) | 3.05(0.1) |
| Acute lymphocytic leukaemia recurrent | 3 | 8.17(2.62-25.46) | 8.17(18.71) | 8.11(2.6) | 3.02(0.09) |
| Skin toxicity | 8 | 7.69(3.83-15.42) | 7.68(46.12) | 7.63(3.8) | 2.93(1.17) |
| Malignant neoplasm progression | 92 | 7.19(5.85-8.84) | 7.11(480.11) | 7.06(5.74) | 2.82(2.43) |
| Hospitalisation | 65 | 7.11(5.56-9.09) | 7.05(335.58) | 7.01(5.48) | 2.81(2.32) |
| Pneumonia fungal | 5 | 7.03(2.92-16.96) | 7.03(25.67) | 6.98(2.9) | 2.8(0.62) |
| Therapy cessation | 40 | 7(5.12-9.56) | 6.96(202.94) | 6.92(5.07) | 2.79(2.14) |
| Acute lymphocytic leukaemia | 3 | 6.83(2.19-21.27) | 6.83(14.82) | 6.79(2.18) | 2.76(0.02) |
| Transfusion | 5 | 6.71(2.78-16.19) | 6.71(24.12) | 6.67(2.77) | 2.74(0.59) |
| Adverse event | 27 | 6.53(4.47-9.54) | 6.51(125.09) | 6.47(4.43) | 2.69(1.89) |
| Insurance issue | 6 | 6.47(2.9-14.44) | 6.46(27.52) | 6.43(2.88) | 2.68(0.76) |
| Lower gastrointestinal haemorrhage | 4 | 6.38(2.38-17.05) | 6.37(18) | 6.34(2.37) | 2.66(0.32) |
| Periorbital oedema | 5 | 5.7(2.37-13.74) | 5.7(19.25) | 5.67(2.35) | 2.5(0.49) |
| Normal newborn | 5 | 5.67(2.35-13.66) | 5.66(19.09) | 5.64(2.34) | 2.49(0.49) |
| Lung infiltration | 11 | 5.66(3.13-10.25) | 5.65(41.9) | 5.63(3.11) | 2.49(1.18) |
| Drug resistance | 16 | 5.62(3.43-9.18) | 5.6(60.19) | 5.58(3.41) | 2.48(1.43) |
| Bone pain | 30 | 5.5(3.84-7.87) | 5.48(109.2) | 5.45(3.8) | 2.45(1.73) |
| Cytopenia | 8 | 5.37(2.68-10.77) | 5.37(28.28) | 5.34(2.67) | 2.42(0.88) |
| Splenomegaly | 10 | 5.27(2.83-9.82) | 5.27(34.38) | 5.24(2.81) | 2.39(1.05) |
| Stress cardiomyopathy | 5 | 5(2.08-12.05) | 5(15.91) | 4.98(2.07) | 2.32(0.4) |
| Nephrotic syndrome | 6 | 4.85(2.17-10.82) | 4.84(18.22) | 4.83(2.16) | 2.27(0.55) |
| Haematotoxicity | 6 | 4.81(2.16-10.73) | 4.81(18.01) | 4.79(2.15) | 2.26(0.54) |
| Pulmonary congestion | 8 | 4.63(2.31-9.27) | 4.62(22.61) | 4.6(2.3) | 2.2(0.75) |
| Clostridium difficile colitis | 7 | 4.57(2.17-9.61) | 4.57(19.41) | 4.55(2.16) | 2.19(0.63) |
| Generalised oedema | 8 | 4.24(2.11-8.49) | 4.23(19.66) | 4.22(2.1) | 2.08(0.67) |
| Skin disorder | 12 | 3.96(2.24-6.98) | 3.95(26.37) | 3.94(2.23) | 1.98(0.88) |
| Dyspnoea exertional | 16 | 3.86(2.36-6.31) | 3.85(33.68) | 3.84(2.35) | 1.94(1.02) |
| Alopecia | 53 | 3.8(2.9-4.98) | 3.78(108.16) | 3.77(2.87) | 1.91(1.45) |
| Oedema | 25 | 3.59(2.42-5.32) | 3.58(46.34) | 3.57(2.41) | 1.84(1.13) |
| Eye swelling | 12 | 3.55(2.01-6.26) | 3.55(21.86) | 3.54(2.01) | 1.82(0.76) |
| Gastrointestinal haemorrhage | 24 | 3.47(2.32-5.18) | 3.46(41.84) | 3.45(2.31) | 1.79(1.07) |
| Headache | 194 | 3.02(2.62-3.48) | 2.96(253.5) | 2.95(2.56) | 1.56(1.34) |
| Fatigue | 192 | 2.94(2.54-3.39) | 2.88(237.5) | 2.88(2.49) | 1.52(1.3) |
| Death | 145 | 2.73(2.31-3.21) | 2.69(154.7) | 2.68(2.28) | 1.42(1.17) |
| Rash | 138 | 2.59(2.19-3.07) | 2.56(131.96) | 2.56(2.16) | 1.35(1.09) |

Abbreviations: PT, Preferred Term; ROR, Reporting Odds Ratio; PRR, Proportional Reporting Ratio; EBGM, Empirical Bayes Geometric Mean; EBGM05, lower 5% one-sided confidence limit of EBGM; IC, Information Component; IC025, lower end of the 95% credibility interval of IC.

**Supplementary Table 7** Distribution of AEs according to PT aged 65-85.

| PT | Case number | ROR(95%Cl) | PRR(XX) | EBGM(EBGM05) | IC(IC025) |
| --- | --- | --- | --- | --- | --- |
| Primary effusion lymphoma | 4 | 441.6(135.93-1434.63) | 441.12(1216.03) | 305.7(94.1) | 8.26(0.8) |
| Chylothorax | 16 | 295.36(168.91-516.48) | 294.08(3605.21) | 227.09(129.87) | 7.83(3.21) |
| Philadelphia chromosome positive | 6 | 96.21(41.59-222.54) | 96.05(514.58) | 87.66(37.9) | 6.45(1.56) |
| Cytogenetic analysis abnormal | 6 | 93.2(40.34-215.34) | 93.05(499.54) | 85.16(36.86) | 6.41(1.56) |
| Aspiration pleural cavity | 6 | 89.03(38.59-205.36) | 88.88(478.52) | 81.66(35.4) | 6.35(1.56) |
| Acute lymphocytic leukaemia recurrent | 7 | 87.01(40.16-188.53) | 86.85(546.22) | 79.94(36.89) | 6.32(1.81) |
| Polyserositis | 3 | 64.78(20.14-208.39) | 64.73(176.71) | 60.83(18.91) | 5.93(0.43) |
| Gene mutation | 3 | 41.39(13.03-131.42) | 41.35(113.41) | 39.74(12.52) | 5.31(0.41) |
| Pleural effusion | 282 | 37.82(33.43-42.79) | 34.99(9013.68) | 33.83(29.9) | 5.08(4.74) |
| Prescribed underdose | 33 | 26.26(18.56-37.16) | 26.04(774.42) | 25.4(17.95) | 4.67(3.38) |
| Lymphocytosis | 6 | 25.27(11.23-56.86) | 25.23(136.19) | 24.63(10.95) | 4.62(1.38) |
| Cytomegalovirus colitis | 4 | 21.37(7.93-57.56) | 21.34(75.94) | 20.92(7.76) | 4.39(0.76) |
| Pericardial effusion | 43 | 19.71(14.55-26.7) | 19.49(740.11) | 19.13(14.12) | 4.26(3.32) |
| Hair texture abnormal | 3 | 18.74(5.98-58.76) | 18.73(49.41) | 18.4(5.87) | 4.2(0.32) |
| Pleurisy | 10 | 18.13(9.69-33.91) | 18.08(158.48) | 17.77(9.5) | 4.15(1.93) |
| Chronic myeloid leukaemia | 5 | 17.25(7.12-41.79) | 17.23(75.15) | 16.95(7) | 4.08(1.02) |
| Pulmonary oedema | 68 | 17.05(13.39-21.71) | 16.75(991.55) | 16.49(12.95) | 4.04(3.4) |
| Fluid retention | 36 | 14.51(10.43-20.2) | 14.38(442.04) | 14.19(10.19) | 3.83(2.91) |
| Periorbital oedema | 5 | 14.28(5.9-34.54) | 14.26(60.78) | 14.07(5.82) | 3.81(0.96) |
| Cytomegalovirus infection reactivation | 3 | 11.78(3.77-36.78) | 11.77(29.22) | 11.64(3.73) | 3.54(0.21) |
| Therapy change | 7 | 11.03(5.23-23.25) | 11.01(63.02) | 10.9(5.17) | 3.45(1.26) |
| Pulmonary hypertension | 23 | 10.7(7.09-16.16) | 10.64(198.92) | 10.54(6.98) | 3.4(2.32) |
| Optic neuritis | 3 | 9.14(2.93-28.5) | 9.13(21.53) | 9.06(2.91) | 3.18(0.13) |
| Ovarian cancer | 3 | 8.08(2.59-25.16) | 8.07(18.43) | 8.01(2.57) | 3(0.09) |
| Polyp | 3 | 8.05(2.58-25.1) | 8.05(18.37) | 7.99(2.56) | 3(0.09) |
| Hyperuricaemia | 5 | 7.75(3.21-18.7) | 7.74(29.13) | 7.69(3.19) | 2.94(0.68) |
| Adverse event | 16 | 7.53(4.6-12.33) | 7.5(89.53) | 7.45(4.55) | 2.9(1.73) |
| Irritable bowel syndrome | 3 | 7.21(2.32-22.47) | 7.21(15.93) | 7.16(2.3) | 2.84(0.04) |
| Pulmonary arterial hypertension | 8 | 6.46(3.22-12.95) | 6.44(36.58) | 6.41(3.2) | 2.68(1.03) |
| Therapy cessation | 15 | 5.78(3.48-9.61) | 5.76(58.7) | 5.73(3.45) | 2.52(1.42) |
| Colitis ulcerative | 6 | 5.73(2.57-12.79) | 5.72(23.25) | 5.69(2.55) | 2.51(0.67) |
| Photosensitivity reaction | 5 | 5.27(2.19-12.71) | 5.27(17.2) | 5.25(2.18) | 2.39(0.44) |
| Transfusion | 5 | 5.22(2.17-12.57) | 5.21(16.94) | 5.19(2.15) | 2.38(0.43) |
| Skin disorder | 8 | 4.88(2.43-9.78) | 4.87(24.5) | 4.85(2.42) | 2.28(0.8) |
| Pulmonary congestion | 5 | 4.86(2.02-11.7) | 4.85(15.22) | 4.83(2.01) | 2.27(0.38) |
| Product dispensing error | 6 | 4.82(2.16-10.75) | 4.81(18.03) | 4.79(2.15) | 2.26(0.54) |
| Malignant neoplasm progression | 44 | 3.91(2.9-5.27) | 3.87(93.75) | 3.86(2.87) | 1.95(1.43) |
| Drug intolerance | 19 | 3.88(2.47-6.09) | 3.86(40.18) | 3.85(2.45) | 1.94(1.1) |
| Hospitalisation | 19 | 3.77(2.4-5.93) | 3.76(38.39) | 3.75(2.39) | 1.91(1.07) |
| Oedema | 18 | 3.38(2.13-5.38) | 3.37(29.94) | 3.36(2.11) | 1.75(0.91) |
| Respiratory failure | 30 | 3.11(2.17-4.46) | 3.09(42.52) | 3.09(2.15) | 1.63(1.01) |
| Fatigue | 91 | 2.55(2.07-3.14) | 2.51(83.13) | 2.5(2.03) | 1.32(1) |

Abbreviations: PT, Preferred Term; ROR, Reporting Odds Ratio; PRR, Proportional Reporting Ratio; EBGM, Empirical Bayes Geometric Mean; EBGM05, lower 5% one-sided confidence limit of EBGM; IC, Information Component; IC025, lower end of the 95% credibility interval of IC.

**Supplementary Table 8** Distribution of AEs according to PT aged >85.

| PT | Case number | ROR(95%Cl) | PRR(XX) | EBGM(EBGM05) | IC(IC025) |
| --- | --- | --- | --- | --- | --- |
| Blast cell proliferation | 6 | 945.53(304.87-2932.49) | 944.7(2828.11) | 472.85(152.46) | 8.89(1.43) |
| Chylothorax | 19 | 243.23(146.85-402.89) | 242.56(3636.95) | 193.21(116.64) | 7.59(3.47) |
| Philadelphia chromosome positive | 12 | 153.46(83.35-282.56) | 153.19(1561.26) | 131.96(71.67) | 7.04(2.71) |
| Aspiration pleural cavity | 8 | 109.66(52.72-228.1) | 109.53(770.94) | 98.25(47.24) | 6.62(2.04) |
| Chronic myeloid leukaemia transformation | 5 | 78.78(31.63-196.24) | 78.73(354.18) | 72.75(29.2) | 6.18(1.26) |
| Lymphoid tissue hyperplasia | 6 | 70.91(30.92-162.62) | 70.85(384.38) | 65.98(28.77) | 6.04(1.55) |
| Cytogenetic analysis abnormal | 9 | 55.64(28.4-109.03) | 55.57(455.52) | 52.54(26.81) | 5.72(2.15) |
| Bone marrow transplant | 9 | 50.37(25.75-98.54) | 50.31(412.98) | 47.82(24.45) | 5.58(2.13) |
| Blast crisis in myelogenous leukaemia | 7 | 42.16(19.77-89.93) | 42.12(269.02) | 40.37(18.92) | 5.34(1.72) |
| Neutrophilic dermatosis | 3 | 38.84(12.24-123.26) | 38.82(106.18) | 37.33(11.76) | 5.22(0.41) |
| Pleural effusion | 283 | 32.46(28.76-36.63) | 31.15(8005.64) | 30.19(26.75) | 4.92(4.6) |
| Acquired gene mutation | 4 | 32.32(11.93-87.57) | 32.3(117.31) | 31.26(11.54) | 4.97(0.83) |
| Transplant | 5 | 27.97(11.49-68.09) | 27.95(126.2) | 27.18(11.16) | 4.76(1.14) |
| Enterocolitis haemorrhagic | 9 | 23.32(12.03-45.21) | 23.29(187.42) | 22.76(11.74) | 4.51(1.91) |
| Gene mutation | 5 | 23.06(9.49-56.01) | 23.04(102.92) | 22.52(9.27) | 4.49(1.1) |
| Right ventricular dysfunction | 4 | 21.24(7.88-57.24) | 21.23(75.41) | 20.78(7.71) | 4.38(0.76) |
| Prescribed underdose | 30 | 19.63(13.66-28.2) | 19.55(517.32) | 19.17(13.34) | 4.26(3.07) |
| Hyperplasia | 3 | 17.72(5.65-55.54) | 17.71(46.44) | 17.41(5.55) | 4.12(0.31) |
| Cytomegalovirus enterocolitis | 3 | 17.72(5.65-55.54) | 17.71(46.44) | 17.41(5.55) | 4.12(0.31) |
| Chronic myeloid leukaemia | 12 | 17.28(9.76-30.61) | 17.25(180.48) | 16.96(9.58) | 4.08(2.12) |
| Lymphocytosis | 8 | 16.96(8.43-34.15) | 16.95(117.93) | 16.66(8.28) | 4.06(1.63) |
| Fluid retention | 54 | 15.42(11.78-20.2) | 15.31(711.14) | 15.08(11.51) | 3.91(3.19) |
| Haemothorax | 6 | 15.21(6.79-34.08) | 15.2(78.32) | 14.97(6.68) | 3.9(1.22) |
| Pleurisy | 16 | 14.13(8.62-23.16) | 14.1(191.9) | 13.91(8.48) | 3.8(2.28) |
| Therapy change | 18 | 13.87(8.7-22.1) | 13.84(211.3) | 13.65(8.57) | 3.77(2.37) |
| Pericardial effusion | 49 | 13.61(10.25-18.06) | 13.52(560.17) | 13.34(10.05) | 3.74(3.01) |
| Pulmonary hypertension | 35 | 12.97(9.29-18.12) | 12.91(379.56) | 12.75(9.13) | 3.67(2.78) |
| Cytomegalovirus colitis | 5 | 12.77(5.28-30.88) | 12.77(53.5) | 12.61(5.22) | 3.66(0.91) |
| Full blood count decreased | 16 | 12.09(7.38-19.81) | 12.06(160.32) | 11.92(7.28) | 3.58(2.15) |
| Stem cell transplant | 5 | 11.64(4.82-28.13) | 11.63(48.01) | 11.5(4.76) | 3.52(0.88) |
| Demyelinating polyneuropathy | 3 | 11.57(3.71-36.14) | 11.57(28.61) | 11.44(3.66) | 3.52(0.21) |
| Pulmonary arterial hypertension | 19 | 10.17(6.47-16) | 10.15(155.04) | 10.05(6.39) | 3.33(2.14) |
| Drug tolerance decreased | 3 | 9.68(3.1-30.18) | 9.67(23.09) | 9.58(3.07) | 3.26(0.15) |
| Sunburn | 5 | 9.21(3.82-22.24) | 9.21(36.23) | 9.13(3.78) | 3.19(0.77) |
| Eye haemorrhage | 7 | 9.02(4.28-18.99) | 9.01(49.38) | 8.93(4.24) | 3.16(1.14) |
| Internal haemorrhage | 3 | 8.86(2.84-27.62) | 8.86(20.72) | 8.78(2.82) | 3.13(0.12) |
| Graft versus host disease in gastrointestinal tract | 3 | 8.75(2.81-27.28) | 8.75(20.4) | 8.68(2.78) | 3.12(0.12) |
| Pulmonary oedema | 44 | 8.57(6.36-11.54) | 8.52(289.64) | 8.45(6.28) | 3.08(2.42) |
| Acne | 26 | 8.56(5.81-12.6) | 8.53(171.32) | 8.46(5.75) | 3.08(2.17) |
| Graft versus host disease | 11 | 8.45(4.66-15.3) | 8.43(71.46) | 8.37(4.62) | 3.07(1.54) |
| Gingival disorder | 3 | 8.36(2.68-26.07) | 8.36(19.27) | 8.3(2.66) | 3.05(0.1) |
| Acute lymphocytic leukaemia recurrent | 3 | 8.17(2.62-25.46) | 8.17(18.71) | 8.11(2.6) | 3.02(0.09) |
| Skin toxicity | 8 | 7.69(3.83-15.42) | 7.68(46.12) | 7.63(3.8) | 2.93(1.17) |
| Malignant neoplasm progression | 92 | 7.19(5.85-8.84) | 7.11(480.11) | 7.06(5.74) | 2.82(2.43) |
| Hospitalisation | 65 | 7.11(5.56-9.09) | 7.05(335.58) | 7.01(5.48) | 2.81(2.32) |
| Pneumonia fungal | 5 | 7.03(2.92-16.96) | 7.03(25.67) | 6.98(2.9) | 2.8(0.62) |
| Therapy cessation | 40 | 7(5.12-9.56) | 6.96(202.94) | 6.92(5.07) | 2.79(2.14) |
| Acute lymphocytic leukaemia | 3 | 6.83(2.19-21.27) | 6.83(14.82) | 6.79(2.18) | 2.76(0.02) |
| Transfusion | 5 | 6.71(2.78-16.19) | 6.71(24.12) | 6.67(2.77) | 2.74(0.59) |
| Adverse event | 27 | 6.53(4.47-9.54) | 6.51(125.09) | 6.47(4.43) | 2.69(1.89) |
| Insurance issue | 6 | 6.47(2.9-14.44) | 6.46(27.52) | 6.43(2.88) | 2.68(0.76) |
| Lower gastrointestinal haemorrhage | 4 | 6.38(2.38-17.05) | 6.37(18) | 6.34(2.37) | 2.66(0.32) |
| Periorbital oedema | 5 | 5.7(2.37-13.74) | 5.7(19.25) | 5.67(2.35) | 2.5(0.49) |
| Normal newborn | 5 | 5.67(2.35-13.66) | 5.66(19.09) | 5.64(2.34) | 2.49(0.49) |
| Lung infiltration | 11 | 5.66(3.13-10.25) | 5.65(41.9) | 5.63(3.11) | 2.49(1.18) |
| Drug resistance | 16 | 5.62(3.43-9.18) | 5.6(60.19) | 5.58(3.41) | 2.48(1.43) |
| Bone pain | 30 | 5.5(3.84-7.87) | 5.48(109.2) | 5.45(3.8) | 2.45(1.73) |
| Cytopenia | 8 | 5.37(2.68-10.77) | 5.37(28.28) | 5.34(2.67) | 2.42(0.88) |
| Splenomegaly | 10 | 5.27(2.83-9.82) | 5.27(34.38) | 5.24(2.81) | 2.39(1.05) |
| Stress cardiomyopathy | 5 | 5(2.08-12.05) | 5(15.91) | 4.98(2.07) | 2.32(0.4) |
| Nephrotic syndrome | 6 | 4.85(2.17-10.82) | 4.84(18.22) | 4.83(2.16) | 2.27(0.55) |
| Haematotoxicity | 6 | 4.81(2.16-10.73) | 4.81(18.01) | 4.79(2.15) | 2.26(0.54) |
| Pulmonary congestion | 8 | 4.63(2.31-9.27) | 4.62(22.61) | 4.6(2.3) | 2.2(0.75) |
| Clostridium difficile colitis | 7 | 4.57(2.17-9.61) | 4.57(19.41) | 4.55(2.16) | 2.19(0.63) |
| Generalised oedema | 8 | 4.24(2.11-8.49) | 4.23(19.66) | 4.22(2.1) | 2.08(0.67) |
| Skin disorder | 12 | 3.96(2.24-6.98) | 3.95(26.37) | 3.94(2.23) | 1.98(0.88) |
| Dyspnoea exertional | 16 | 3.86(2.36-6.31) | 3.85(33.68) | 3.84(2.35) | 1.94(1.02) |
| Alopecia | 53 | 3.8(2.9-4.98) | 3.78(108.16) | 3.77(2.87) | 1.91(1.45) |
| Oedema | 25 | 3.59(2.42-5.32) | 3.58(46.34) | 3.57(2.41) | 1.84(1.13) |
| Eye swelling | 12 | 3.55(2.01-6.26) | 3.55(21.86) | 3.54(2.01) | 1.82(0.76) |
| Gastrointestinal haemorrhage | 24 | 3.47(2.32-5.18) | 3.46(41.84) | 3.45(2.31) | 1.79(1.07) |
| Headache | 194 | 3.02(2.62-3.48) | 2.96(253.5) | 2.95(2.56) | 1.56(1.34) |
| Fatigue | 192 | 2.94(2.54-3.39) | 2.88(237.5) | 2.88(2.49) | 1.52(1.3) |
| Death | 145 | 2.73(2.31-3.21) | 2.69(154.7) | 2.68(2.28) | 1.42(1.17) |
| Rash | 138 | 2.59(2.19-3.07) | 2.56(131.96) | 2.56(2.16) | 1.35(1.09) |

Abbreviations: PT, Preferred Term; ROR, Reporting Odds Ratio; PRR, Proportional Reporting Ratio; EBGM, Empirical Bayes Geometric Mean; EBGM05, lower 5% one-sided confidence limit of EBGM; IC, Information Component; IC025, lower end of the 95% credibility interval of IC.

**Supplementary Table 9** Distribution of AEs according to PT weighting <50kg.

| PT | Case number | ROR(95%Cl) | PRR(XX) | EBGM(EBGM05) | IC(IC025) |
| --- | --- | --- | --- | --- | --- |
| Prescribed underdose | 3 | 43.15(13.69-135.98) | 42.63(120.04) | 41.96(13.32) | 5.39(0.43) |
| Pleural effusion | 18 | 32.78(20.23-53.11) | 30.42(507.56) | 30.08(18.57) | 4.91(2.88) |
| Pulmonary hypertension | 3 | 14.75(4.71-46.2) | 14.58(37.77) | 14.5(4.63) | 3.86(0.27) |
| Encephalopathy | 3 | 12.63(4.04-39.56) | 12.49(31.59) | 12.44(3.97) | 3.64(0.23) |
| Gastrointestinal haemorrhage | 4 | 9.51(3.53-25.6) | 9.37(29.86) | 9.34(3.47) | 3.22(0.5) |
| Weight increased | 3 | 8.72(2.79-27.29) | 8.63(20.2) | 8.6(2.75) | 3.11(0.11) |
| Malignant neoplasm progression | 5 | 7.65(3.15-18.56) | 7.51(28.21) | 7.49(3.09) | 2.91(0.65) |
| Cardiac failure | 5 | 6.62(2.73-16.06) | 6.5(23.29) | 6.49(2.67) | 2.7(0.56) |

Abbreviations: PT, Preferred Term; ROR, Reporting Odds Ratio; PRR, Proportional Reporting Ratio; EBGM, Empirical Bayes Geometric Mean; EBGM05, lower 5% one-sided confidence limit of EBGM; IC, Information Component; IC025, lower end of the 95% credibility interval of IC.

**Supplementary Table 10** Distribution of AEs according to PT weighing 50-100kg.

| PT | Case number | ROR(95%Cl) | PRR(XX) | EBGM(EBGM05) | IC(IC025) |
| --- | --- | --- | --- | --- | --- |
| Chylothorax | 11 | 540.98(278.06-1052.5) | 537.51(4668.14) | 426.16(219.04) | 8.74(2.62) |
| Pleural effusion | 109 | 32.28(26.55-39.24) | 30.29(3048.96) | 29.87(24.57) | 4.9(4.28) |
| Pulmonary hypertension | 20 | 23.29(14.95-36.29) | 23.03(417.08) | 22.79(14.63) | 4.51(2.85) |
| Chronic myeloid leukaemia | 3 | 21.87(7-68.29) | 21.83(59.01) | 21.61(6.92) | 4.43(0.36) |
| Pericardial effusion | 22 | 20.18(13.23-30.79) | 19.93(392.11) | 19.75(12.94) | 4.3(2.83) |
| Stress cardiomyopathy | 4 | 19.5(7.28-52.25) | 19.45(69.37) | 19.28(7.19) | 4.27(0.75) |
| Rectal cancer | 3 | 19.33(6.2-60.32) | 19.3(51.58) | 19.13(6.13) | 4.26(0.34) |
| Periorbital oedema | 3 | 17.37(5.57-54.18) | 17.34(45.82) | 17.21(5.52) | 4.1(0.32) |
| Prescribed underdose | 8 | 16.57(8.25-33.29) | 16.5(115.6) | 16.38(8.15) | 4.03(1.63) |
| Drug resistance | 4 | 16.52(6.17-44.24) | 16.48(57.73) | 16.36(6.11) | 4.03(0.71) |
| Haemothorax | 3 | 16.4(5.26-51.14) | 16.37(42.97) | 16.25(5.21) | 4.02(0.3) |
| Pleurisy | 5 | 15.81(6.55-38.15) | 15.76(68.62) | 15.65(6.48) | 3.97(1) |
| Pulmonary arterial hypertension | 8 | 15.65(7.79-31.42) | 15.58(108.34) | 15.47(7.7) | 3.95(1.6) |
| Product storage error | 6 | 14.83(6.63-33.15) | 14.78(76.57) | 14.68(6.57) | 3.88(1.22) |
| Pulmonary oedema | 19 | 11.78(7.49-18.54) | 11.66(184.32) | 11.6(7.37) | 3.54(2.27) |
| Lung infiltration | 8 | 11.42(5.69-22.91) | 11.37(75.28) | 11.31(5.64) | 3.5(1.43) |
| Right ventricular failure | 4 | 10.74(4.02-28.72) | 10.72(35.07) | 10.67(3.99) | 3.42(0.57) |
| Cardiac failure chronic | 3 | 10.58(3.4-32.93) | 10.56(25.84) | 10.51(3.38) | 3.39(0.19) |
| Optic neuritis | 3 | 10.35(3.32-32.2) | 10.33(25.16) | 10.28(3.3) | 3.36(0.18) |
| Therapy interrupted | 4 | 10.02(3.75-26.79) | 10(32.25) | 9.96(3.72) | 3.32(0.54) |
| Hyperuricaemia | 3 | 8.36(2.69-25.99) | 8.34(19.31) | 8.31(2.67) | 3.06(0.11) |
| Fluid retention | 8 | 8.23(4.11-16.52) | 8.2(50.41) | 8.17(4.07) | 3.03(1.22) |
| Cytopenia | 3 | 7.44(2.39-23.13) | 7.43(16.63) | 7.4(2.38) | 2.89(0.06) |
| Eyelid oedema | 3 | 7.11(2.29-22.12) | 7.1(15.68) | 7.08(2.28) | 2.82(0.04) |
| Generalised oedema | 4 | 6.78(2.54-18.12) | 6.77(19.6) | 6.75(2.53) | 2.75(0.35) |
| Malignant neoplasm progression | 17 | 5.4(3.35-8.71) | 5.36(60.2) | 5.35(3.31) | 2.42(1.42) |
| Dyspnoea exertional | 8 | 4.81(2.4-9.64) | 4.79(23.98) | 4.78(2.39) | 2.26(0.79) |
| Respiratory failure | 14 | 3.86(2.28-6.54) | 3.84(29.42) | 3.84(2.27) | 1.94(0.94) |
| Pancytopenia | 14 | 3.68(2.17-6.23) | 3.66(27.04) | 3.65(2.16) | 1.87(0.88) |

Abbreviations: PT, Preferred Term; ROR, Reporting Odds Ratio; PRR, Proportional Reporting Ratio; EBGM, Empirical Bayes Geometric Mean; EBGM05, lower 5% one-sided confidence limit of EBGM; IC, Information Component; IC025, lower end of the 95% credibility interval of IC.

**Supplementary Table 11** Distribution of AEs according to PT weighing >100kg.

| PT | Case number | ROR(95%Cl) | PRR(XX) | EBGM(EBGM05) | IC(IC025) |
| --- | --- | --- | --- | --- | --- |
| Pleural effusion | 23 | 50.71(33.04-77.85) | 47.36(1017.34) | 46.12(30.04) | 5.53(3.38) |
| Fluid retention | 8 | 24.24(11.96-49.1) | 23.69(171.68) | 23.38(11.54) | 4.55(1.76) |
| Pericardial effusion | 4 | 19.64(7.29-52.92) | 19.42(69.15) | 19.21(7.13) | 4.26(0.74) |
| Dyspnoea exertional | 4 | 8.52(3.17-22.88) | 8.43(26.09) | 8.39(3.12) | 3.07(0.45) |
| Pulmonary oedema | 3 | 7.65(2.45-23.9) | 7.59(17.12) | 7.56(2.42) | 2.92(0.06) |
| Headache | 11 | 4.22(2.31-7.7) | 4.11(26.07) | 4.11(2.25) | 2.04(0.85) |

Abbreviations: PT, Preferred Term; ROR, Reporting Odds Ratio; PRR, Proportional Reporting Ratio; EBGM, Empirical Bayes Geometric Mean; EBGM05, lower 5% one-sided confidence limit of EBGM; IC, Information Component; IC025, lower end of the 95% credibility interval of IC.

**Supplementary Table 12** Sensitivity analysis of adverse event signals stratified by reporter type (physician): Top 30 preferred terms.

| PT | Case number | ROR(95%Cl) | PRR(XX) | EBGM(EBGM05) | IC(IC025) |
| --- | --- | --- | --- | --- | --- |
| Pleural effusion | 567 | 38.81(35.6-42.3) | 36.48(19014.65) | 35.42(32.5) | 5.15(4.93) |
| Hepatotoxicity | 262 | 47.68(42.07-54.04) | 46.35(11195.63) | 44.65(39.39) | 5.48(5.07) |
| Death | 248 | 2.08(1.84-2.36) | 2.06(135.91) | 2.05(1.81) | 1.04(0.85) |
| Fatigue | 179 | 2.22(1.92-2.58) | 2.2(118.11) | 2.2(1.9) | 1.14(0.91) |
| Malignant neoplasm progression | 166 | 6.74(5.78-7.87) | 6.64(793.16) | 6.61(5.67) | 2.72(2.45) |
| Diarrhoea | 157 | 1.91(1.63-2.24) | 1.9(66.88) | 1.89(1.62) | 0.92(0.68) |
| Nausea | 154 | 1.68(1.43-1.97) | 1.67(41.34) | 1.66(1.42) | 0.74(0.5) |
| Dyspnoea | 135 | 1.76(1.49-2.09) | 1.75(43.87) | 1.75(1.48) | 0.81(0.55) |
| Headache | 133 | 2.01(1.69-2.39) | 2(66.53) | 1.99(1.68) | 1(0.73) |
| Rash | 130 | 1.86(1.57-2.21) | 1.85(51.01) | 1.85(1.55) | 0.89(0.62) |
| Anaemia | 102 | 2.19(1.8-2.66) | 2.18(65.27) | 2.18(1.79) | 1.12(0.82) |
| Pyrexia | 93 | 1.37(1.12-1.69) | 1.37(9.35) | 1.37(1.12) | 0.45(0.15) |
| Off label use | 90 | 0.65(0.52-0.79) | 0.65(17.33) | 0.65(0.53) | -0.62(-0.92) |
| Thrombocytopenia | 82 | 2.55(2.05-3.17) | 2.54(76.33) | 2.53(2.04) | 1.34(1) |
| Drug intolerance | 79 | 4.48(3.59-5.59) | 4.45(210.62) | 4.43(3.55) | 2.15(1.76) |
| Pneumonia | 74 | 1.22(0.97-1.53) | 1.22(2.93) | 1.22(0.97) | 0.29(-0.05) |
| Vomiting | 67 | 1.04(0.81-1.32) | 1.04(0.08) | 1.04(0.81) | 0.05(-0.3) |
| Pericardial effusion | 65 | 12.32(9.64-15.74) | 12.24(664.38) | 12.12(9.49) | 3.6(3.02) |
| Adverse event | 61 | 7.14(5.55-9.2) | 7.1(318.28) | 7.07(5.49) | 2.82(2.32) |
| Prescribed underdose | 59 | 22.04(17.02-28.53) | 21.9(1155.87) | 21.52(16.62) | 4.43(3.63) |
| Pancytopenia | 56 | 3.3(2.54-4.3) | 3.29(89.19) | 3.28(2.52) | 1.72(1.27) |
| Pulmonary hypertension | 56 | 12.19(9.36-15.87) | 12.12(565.93) | 12.01(9.22) | 3.59(2.95) |
| Platelet count decreased | 56 | 2.37(1.82-3.09) | 2.36(44.13) | 2.36(1.82) | 1.24(0.82) |
| Decreased appetite | 55 | 1.66(1.27-2.16) | 1.65(14.25) | 1.65(1.27) | 0.72(0.32) |
| Pulmonary oedema | 54 | 6.92(5.29-9.05) | 6.89(270.4) | 6.85(5.24) | 2.78(2.24) |
| Arthralgia | 53 | 0.91(0.69-1.19) | 0.91(0.49) | 0.91(0.69) | -0.14(-0.53) |
| Drug ineffective | 53 | 0.39(0.29-0.5) | 0.39(51.73) | 0.39(0.3) | -1.36(-1.74) |
| Hospitalisation | 52 | 3.91(2.97-5.13) | 3.89(111.43) | 3.88(2.95) | 1.96(1.48) |
| Weight decreased | 49 | 1.46(1.1-1.93) | 1.46(7.04) | 1.46(1.1) | 0.54(0.12) |
| Fluid retention | 49 | 10.73(8.09-14.22) | 10.68(426.12) | 10.59(7.99) | 3.4(2.74) |

Abbreviations: PT, Preferred Term; ROR, Reporting Odds Ratio; PRR, Proportional Reporting Ratio; EBGM, Empirical Bayes Geometric Mean; EBGM05, lower 5% one-sided confidence limit of EBGM; IC, Information Component; IC025, lower end of the 95% credibility interval of IC.

**Supplementary Table 13** Sensitivity analysis of adverse event signals stratified by reporter type (pharmacist): Top 30 preferred terms.

| PT | Case number | ROR(95%Cl) | PRR(XX) | EBGM(EBGM05) | IC(IC025) |
| --- | --- | --- | --- | --- | --- |
| Fatigue | 198 | 2.64(2.29-3.05) | 2.59(194.35) | 2.58(2.24) | 1.37(1.15) |
| Pleural effusion | 187 | 41.01(35.23-47.74) | 39.7(6477.7) | 36.5(31.36) | 5.19(4.72) |
| Nausea | 183 | 2.1(1.81-2.44) | 2.07(102.08) | 2.06(1.78) | 1.04(0.82) |
| Headache | 172 | 2.96(2.54-3.44) | 2.9(214.87) | 2.89(2.48) | 1.53(1.29) |
| Diarrhoea | 168 | 2.01(1.72-2.34) | 1.98(82.07) | 1.97(1.69) | 0.98(0.75) |
| Rash | 138 | 2.23(1.89-2.65) | 2.2(91.38) | 2.2(1.86) | 1.14(0.88) |
| Hospitalisation | 132 | 4.1(3.45-4.88) | 4.03(299.92) | 4(3.37) | 2(1.71) |
| Death | 123 | 1.37(1.15-1.64) | 1.36(12.13) | 1.36(1.14) | 0.45(0.18) |
| Dyspnoea | 111 | 1.59(1.31-1.91) | 1.57(23.43) | 1.57(1.3) | 0.65(0.37) |
| Pulmonary oedema | 87 | 21.65(17.43-26.88) | 21.33(1609.37) | 20.39(16.42) | 4.35(3.74) |
| Adverse event | 77 | 9.88(7.87-12.41) | 9.77(593.53) | 9.58(7.63) | 3.26(2.78) |
| Off label use | 74 | 1.19(0.94-1.49) | 1.19(2.16) | 1.18(0.94) | 0.24(-0.09) |
| Product storage error | 70 | 2.04(1.61-2.59) | 2.03(36.6) | 2.02(1.6) | 1.02(0.65) |
| Product dose omission issue | 70 | 0.89(0.71-1.13) | 0.89(0.88) | 0.89(0.71) | -0.16(-0.5) |
| Fluid retention | 65 | 14.56(11.36-18.67) | 14.41(786.05) | 13.98(10.91) | 3.81(3.18) |
| Vomiting | 64 | 1.17(0.92-1.5) | 1.17(1.61) | 1.17(0.91) | 0.23(-0.14) |
| Alopecia | 56 | 4.48(3.44-5.84) | 4.45(148.53) | 4.41(3.39) | 2.14(1.67) |
| Arthralgia | 55 | 2.48(1.9-3.24) | 2.47(47.98) | 2.46(1.89) | 1.3(0.87) |
| Drug ineffective | 55 | 0.71(0.55-0.93) | 0.72(6.29) | 0.72(0.55) | -0.48(-0.86) |
| Prescribed underdose | 55 | 37.35(28.33-49.24) | 37(1778.07) | 34.22(25.95) | 5.1(4.02) |
| Pyrexia | 53 | 1.44(1.1-1.89) | 1.44(7.04) | 1.43(1.09) | 0.52(0.11) |
| Pain | 49 | 1.59(1.2-2.11) | 1.59(10.62) | 1.58(1.19) | 0.66(0.24) |
| Therapy cessation | 49 | 3.45(2.6-4.58) | 3.43(83.94) | 3.41(2.57) | 1.77(1.29) |
| Cough | 46 | 2.01(1.5-2.69) | 2(22.97) | 1.99(1.49) | 1(0.54) |
| Pneumonia | 46 | 1.28(0.96-1.71) | 1.28(2.78) | 1.28(0.95) | 0.35(-0.08) |
| Myalgia | 41 | 2.63(1.93-3.58) | 2.62(40.92) | 2.61(1.92) | 1.38(0.88) |
| Chest pain | 41 | 2.01(1.48-2.73) | 2(20.53) | 2(1.47) | 1(0.52) |
| Pruritus | 40 | 0.95(0.7-1.3) | 0.95(0.11) | 0.95(0.7) | -0.07(-0.53) |
| Bone pain | 40 | 8.75(6.39-11.98) | 8.7(267.4) | 8.55(6.24) | 3.1(2.39) |
| Weight decreased | 40 | 2.47(1.81-3.38) | 2.46(34.59) | 2.45(1.8) | 1.29(0.79) |

Abbreviations: PT, Preferred Term; ROR, Reporting Odds Ratio; PRR, Proportional Reporting Ratio; EBGM, Empirical Bayes Geometric Mean; EBGM05, lower 5% one-sided confidence limit of EBGM; IC, Information Component; IC025, lower end of the 95% credibility interval of IC.

**Supplementary Table 14** Sensitivity analysis of adverse event signals stratified by geographic region (United States): Top 30 preferred terms.

| PT | Case number | ROR(95%Cl) | PRR(XX) | EBGM(EBGM05) | IC(IC025) |
| --- | --- | --- | --- | --- | --- |
| Pleural effusion | 465 | 50.02(45.43-55.06) | 47.65(19937.72) | 44.75(40.65) | 5.48(5.21) |
| Fatigue | 337 | 2.82(2.53-3.15) | 2.76(381.76) | 2.75(2.47) | 1.46(1.29) |
| Nausea | 304 | 2.51(2.24-2.82) | 2.46(267.02) | 2.46(2.19) | 1.3(1.12) |
| Headache | 274 | 2.9(2.57-3.27) | 2.84(329.27) | 2.84(2.51) | 1.5(1.32) |
| Diarrhoea | 264 | 2.63(2.33-2.97) | 2.59(258.41) | 2.58(2.28) | 1.37(1.18) |
| Hepatotoxicity | 252 | 99.09(86.74-113.19) | 96.52(21007.17) | 85.21(74.59) | 6.41(5.8) |
| Rash | 237 | 2.38(2.09-2.71) | 2.35(184.34) | 2.34(2.06) | 1.23(1.03) |
| Death | 188 | 1.3(1.13-1.5) | 1.3(12.87) | 1.3(1.12) | 0.37(0.16) |
| Dyspnoea | 177 | 2.05(1.77-2.38) | 2.04(93.77) | 2.03(1.75) | 1.02(0.8) |
| Hospitalisation | 176 | 6.71(5.77-7.79) | 6.6(831.56) | 6.55(5.64) | 2.71(2.45) |
| Adverse event | 136 | 9.7(8.18-11.5) | 9.58(1032.44) | 9.46(7.98) | 3.24(2.91) |
| Pulmonary oedema | 122 | 17.57(14.66-21.05) | 17.36(1837.81) | 16.97(14.17) | 4.09(3.64) |
| Off label use | 111 | 0.73(0.61-0.88) | 0.74(10.69) | 0.74(0.61) | -0.44(-0.71) |
| Product dose omission issue | 107 | 1.02(0.84-1.23) | 1.02(0.04) | 1.02(0.84) | 0.03(-0.25) |
| Vomiting | 100 | 1.49(1.22-1.81) | 1.48(15.67) | 1.48(1.21) | 0.57(0.27) |
| Fluid retention | 97 | 13.21(10.8-16.16) | 13.09(1064.26) | 12.87(10.52) | 3.69(3.23) |
| Malignant neoplasm progression | 91 | 9.68(7.87-11.92) | 9.6(692.67) | 9.49(7.71) | 3.25(2.81) |
| Arthralgia | 85 | 1.17(0.95-1.45) | 1.17(2.09) | 1.17(0.94) | 0.23(-0.09) |
| Alopecia | 78 | 3.3(2.64-4.13) | 3.29(123.8) | 3.28(2.62) | 1.71(1.34) |
| Pneumonia | 78 | 1.43(1.14-1.78) | 1.42(9.85) | 1.42(1.14) | 0.51(0.17) |
| Decreased appetite | 77 | 2.68(2.14-3.36) | 2.67(80.19) | 2.66(2.13) | 1.41(1.05) |
| Product storage error | 76 | 2.55(2.03-3.19) | 2.53(70.57) | 2.53(2.02) | 1.34(0.98) |
| Pain | 73 | 0.94(0.75-1.18) | 0.94(0.29) | 0.94(0.75) | -0.09(-0.43) |
| Cough | 71 | 1.72(1.36-2.18) | 1.72(21.31) | 1.72(1.36) | 0.78(0.42) |
| Prescribed underdose | 71 | 35.05(27.6-44.51) | 34.8(2223.64) | 33.24(26.17) | 5.05(4.17) |
| Pyrexia | 70 | 1.45(1.15-1.84) | 1.45(9.82) | 1.45(1.15) | 0.54(0.18) |
| Anaemia | 69 | 2.19(1.73-2.78) | 2.19(44.42) | 2.18(1.72) | 1.13(0.76) |
| Weight decreased | 67 | 1.9(1.49-2.41) | 1.89(28.12) | 1.89(1.48) | 0.92(0.55) |
| Drug ineffective | 66 | 0.36(0.28-0.46) | 0.36(74.37) | 0.37(0.29) | -1.45(-1.79) |
| Myalgia | 65 | 2.15(1.69-2.75) | 2.14(39.66) | 2.14(1.68) | 1.1(0.72) |

Abbreviations: PT, Preferred Term; ROR, Reporting Odds Ratio; PRR, Proportional Reporting Ratio; EBGM, Empirical Bayes Geometric Mean; EBGM05, lower 5% one-sided confidence limit of EBGM; IC, Information Component; IC025, lower end of the 95% credibility interval of IC.

**Supplementary Table 15** Sensitivity analysis of adverse event signals stratified by geographic region (Japan): Top 30 preferred terms.

| PT | Case number | ROR(95%Cl) | PRR(XX) | EBGM(EBGM05) | IC(IC025) |
| --- | --- | --- | --- | --- | --- |
| Pleural effusion | 71 | 19.91(15.62-25.38) | 18.69(1168.13) | 18.32(14.37) | 4.2(3.53) |
| Anaemia | 23 | 2.44(1.61-3.69) | 2.41(19.08) | 2.41(1.59) | 1.27(0.59) |
| Pyrexia | 22 | 1.36(0.89-2.07) | 1.35(2.03) | 1.35(0.88) | 0.43(-0.2) |
| Prescribed underdose | 22 | 22.82(14.89-34.99) | 22.38(438.78) | 21.86(14.26) | 4.45(2.9) |
| Platelet count decreased | 22 | 1.79(1.17-2.73) | 1.77(7.49) | 1.77(1.16) | 0.83(0.17) |
| Respiratory failure | 14 | 4.48(2.64-7.6) | 4.44(37.21) | 4.42(2.61) | 2.14(1.09) |
| Malignant neoplasm progression | 14 | 1.53(0.9-2.59) | 1.52(2.51) | 1.52(0.9) | 0.6(-0.2) |
| White blood cell count decreased | 14 | 2.02(1.19-3.42) | 2.01(7.1) | 2(1.18) | 1(0.16) |
| Death | 13 | 1.31(0.76-2.26) | 1.31(0.93) | 1.3(0.75) | 0.38(-0.43) |
| Enterocolitis | 13 | 8.82(5.09-15.28) | 8.73(88.21) | 8.65(4.99) | 3.11(1.7) |
| Off label use | 13 | 1.18(0.68-2.04) | 1.18(0.36) | 1.18(0.68) | 0.24(-0.56) |
| Neutrophil count decreased | 13 | 1.98(1.15-3.43) | 1.97(6.25) | 1.97(1.14) | 0.98(0.1) |
| Gastrointestinal haemorrhage | 12 | 4.9(2.77-8.67) | 4.86(36.68) | 4.84(2.74) | 2.27(1.09) |
| Interstitial lung disease | 12 | 0.9(0.51-1.59) | 0.9(0.14) | 0.9(0.51) | -0.15(-0.95) |
| Pneumonia | 11 | 0.9(0.5-1.63) | 0.9(0.12) | 0.9(0.5) | -0.15(-0.98) |
| Fracture | 10 | 3.4(1.82-6.34) | 3.37(16.68) | 3.36(1.8) | 1.75(0.59) |
| Cardiac failure | 10 | 1.32(0.71-2.46) | 1.32(0.77) | 1.32(0.71) | 0.4(-0.52) |
| Rash | 9 | 1.11(0.57-2.13) | 1.11(0.09) | 1.11(0.57) | 0.15(-0.79) |
| Colon cancer | 9 | 5.01(2.6-9.67) | 4.98(28.49) | 4.96(2.57) | 2.31(0.91) |
| Chylothorax | 9 | 183.18(89.21-376.14) | 181.68(1342.65) | 151(73.54) | 7.24(2.24) |
| Pericardial effusion | 9 | 11.08(5.73-21.44) | 11(80.84) | 10.87(5.62) | 3.44(1.53) |
| Enterocolitis haemorrhagic | 9 | 33.72(17.28-65.77) | 33.45(273.09) | 32.27(16.54) | 5.01(2.03) |
| Neoplasm malignant | 8 | 5.54(2.76-11.12) | 5.5(29.34) | 5.48(2.73) | 2.45(0.9) |
| Diarrhoea | 7 | 0.61(0.29-1.28) | 0.61(1.75) | 0.61(0.29) | -0.71(-1.66) |
| Pulmonary hypertension | 7 | 8.16(3.87-17.22) | 8.12(43.32) | 8.05(3.82) | 3.01(1.07) |
| Nausea | 6 | 0.7(0.31-1.56) | 0.7(0.76) | 0.7(0.31) | -0.51(-1.54) |
| Liver disorder | 6 | 0.91(0.41-2.04) | 0.91(0.05) | 0.91(0.41) | -0.13(-1.21) |
| Gastric cancer | 6 | 4.54(2.03-10.16) | 4.52(16.41) | 4.51(2.02) | 2.17(0.49) |
| No adverse event | 6 | 2.81(1.26-6.28) | 2.8(6.94) | 2.79(1.25) | 1.48(0.06) |
| Lung neoplasm malignant | 6 | 3.77(1.69-8.42) | 3.75(12.08) | 3.74(1.67) | 1.9(0.33) |

Abbreviations: PT, Preferred Term; ROR, Reporting Odds Ratio; PRR, Proportional Reporting Ratio; EBGM, Empirical Bayes Geometric Mean; EBGM05, lower 5% one-sided confidence limit of EBGM; IC, Information Component; IC025, lower end of the 95% credibility interval of IC.

**Supplementary Table 16** Sensitivity analysis of adverse event signals stratified by geographic region (France): Top 30 preferred terms.

| PT | Case number | ROR(95%Cl) | PRR(XX) | EBGM(EBGM05) | IC(IC025) |
| --- | --- | --- | --- | --- | --- |
| Pleural effusion | 33 | 43.26(30.27-61.83) | 40.64(1241.65) | 39.51(27.65) | 5.3(3.69) |
| Pleurisy | 10 | 61.86(32.65-117.2) | 60.72(562.93) | 58.22(30.73) | 5.86(2.33) |
| Prescribed underdose | 8 | 58.5(28.69-119.26) | 57.63(427.59) | 55.38(27.16) | 5.79(1.98) |
| Pericardial effusion | 8 | 21.96(10.86-44.37) | 21.64(155.19) | 21.32(10.55) | 4.41(1.73) |
| Thrombocytopenia | 7 | 1.49(0.7-3.13) | 1.48(1.1) | 1.48(0.7) | 0.56(-0.55) |
| Respiratory failure | 7 | 12.73(6.02-26.92) | 12.57(73.99) | 12.47(5.9) | 3.64(1.32) |
| Anaemia | 6 | 1.52(0.68-3.41) | 1.52(1.07) | 1.52(0.68) | 0.6(-0.6) |
| Diarrhoea | 6 | 1.19(0.53-2.67) | 1.19(0.18) | 1.19(0.53) | 0.25(-0.89) |
| Hypertension | 6 | 3.53(1.58-7.91) | 3.5(10.74) | 3.5(1.56) | 1.81(0.26) |
| Dyspnoea | 5 | 1.47(0.61-3.55) | 1.47(0.75) | 1.47(0.61) | 0.55(-0.74) |
| Pyrexia | 5 | 1.16(0.48-2.81) | 1.16(0.11) | 1.16(0.48) | 0.22(-1.01) |
| Chylothorax | 5 | 701.78(239.05-2060.17) | 695.19(2310.69) | 463.79(157.99) | 8.86(1.18) |
| Drug interaction | 5 | 1.19(0.49-2.87) | 1.19(0.15) | 1.19(0.49) | 0.25(-0.99) |
| Cardiac failure | 5 | 2.61(1.08-6.31) | 2.6(4.93) | 2.6(1.08) | 1.38(-0.15) |
| Subdural haematoma | 5 | 10.15(4.19-24.56) | 10.06(40.55) | 10(4.13) | 3.32(0.81) |
| Malignant neoplasm progression | 5 | 4.5(1.86-10.87) | 4.46(13.43) | 4.45(1.84) | 2.16(0.31) |
| Interstitial lung disease | 5 | 2.39(0.99-5.76) | 2.37(3.98) | 2.37(0.98) | 1.25(-0.24) |
| Pulmonary arterial hypertension | 5 | 13.21(5.45-31.99) | 13.09(55.36) | 12.98(5.36) | 3.7(0.92) |
| Fatigue | 4 | 2.14(0.8-5.73) | 2.13(2.41) | 2.13(0.8) | 1.09(-0.5) |
| Vomiting | 4 | 0.85(0.32-2.28) | 0.85(0.1) | 0.85(0.32) | -0.23(-1.49) |
| Neutropenia | 4 | 1.03(0.38-2.75) | 1.03(0) | 1.03(0.38) | 0.04(-1.27) |
| Inflammation | 4 | 6.1(2.28-16.36) | 6.06(16.87) | 6.04(2.25) | 2.6(0.29) |
| Renal failure | 4 | 1.51(0.56-4.04) | 1.51(0.68) | 1.51(0.56) | 0.59(-0.85) |
| Pulmonary hypertension | 4 | 19.38(7.2-52.19) | 19.24(68.27) | 18.99(7.05) | 4.25(0.74) |
| Atrial fibrillation | 4 | 3.58(1.34-9.58) | 3.56(7.35) | 3.55(1.33) | 1.83(-0.07) |
| Dyspnoea exertional | 4 | 12.02(4.47-32.27) | 11.93(39.76) | 11.84(4.41) | 3.57(0.6) |
| Lymphoid tissue hyperplasia | 4 | 622.62(191.15-2028.07) | 617.95(1705.73) | 428.12(131.43) | 8.74(0.8) |
| Fall | 3 | 0.81(0.26-2.53) | 0.82(0.13) | 0.82(0.26) | -0.29(-1.68) |
| Bladder cancer | 3 | 47.13(14.87-149.38) | 46.87(130.29) | 45.37(14.31) | 5.5(0.43) |
| Off label use | 3 | 0.39(0.13-1.22) | 0.4(2.8) | 0.4(0.13) | -1.33(-2.55) |

Abbreviations: PT, Preferred Term; ROR, Reporting Odds Ratio; PRR, Proportional Reporting Ratio; EBGM, Empirical Bayes Geometric Mean; EBGM05, lower 5% one-sided confidence limit of EBGM; IC, Information Component; IC025, lower end of the 95% credibility interval of IC.
